# Critical Review of Natural Fiber Reinforced Hybrid Composites: Processing, Properties, Applications and Cost

**DOI:** 10.3390/polym13203514

**Published:** 2021-10-13

**Authors:** M. J. Suriani, R. A. Ilyas, M. Y. M. Zuhri, A. Khalina, M. T. H. Sultan, S. M. Sapuan, C. M. Ruzaidi, F. Nik Wan, F. Zulkifli, M. M. Harussani, M. A. Azman, F. S. M. Radzi, Shubham Sharma

**Affiliations:** 1 Faculty of Ocean Engineering Technology and Informatics, Universiti Malaysia Terengganu, Kuala Nerus 21030, Terengganu, Malaysia; ruzaidi@umt.edu.my (C.M.R.); wnfatihahnw@gmail.com (F.N.W.); fakhratulz@umt.edu.my (F.Z.); asyrafazman23@yahoo.com (M.A.A.); fathinsakinah96@gmail.com (F.S.M.R.); 2 School of Chemical and Energy Engineering, Faculty of Engineering, Universiti Teknologi Malaysia (UTM), Johor Bahru 81310, Johor, Malaysia; 3 Centre for Advanced Composite Materials (CACM), Universiti Teknologi Malaysia (UTM), Johor Bahru 81310, Johor, Malaysia; 4 Advanced Engineering Materials and Composites Research Centre (AEMC), Department of Mechanical and Manufacturing Engineering, Faculty of Engineering, Universiti Putra Malaysia, Serdang 43400, Selangor, Malaysia; sapuan@upm.edu.my (S.M.S.); mmharussani17@gmail.com (M.M.H.); 5 Laboratory of Biocomposite Technology, Institute of Tropical Forestry and Forest Products (INTROP), Universiti Putra Malaysia, Serdang 43400, Selangor, Malaysia; khalina@upm.edu.my (A.K.); thariq@upm.edu.my (M.T.H.S.); 6 Department of Biological and Agricultural Engineering, Faculty of Engineering, Universiti Putra Malaysia, Serdang 43400, Selangor, Malaysia; 7 Department of Aerospace Engineering, Faculty of Engineering, Universiti Putra Malaysia, Serdang 43400, Selangor, Malaysia; 8 Department of Mechanical Engineering, IK Gujral Punjab Technical University, Main Campus-Kapurthala, Punjab 144603, India; shubham543sharma@gmail.com

**Keywords:** natural fiber, hybrid composite, cellulose, costing, processing, fiber-matrix adhesion

## Abstract

Increasing scientific interest has occurred concerning the utilization of natural fiber-enhanced hybrid composites that incorporate one or more types of natural enhancement. Annual natural fiber production is estimated to be 1,783,965 $\times \;10^{3}$ tons/year. Extensive studies have been conducted in the domains of natural/synthetic as well as natural/natural hybrid composites. As synthetic fibers have better rigidity and strength than natural fibers, natural/synthetic hybrid composites have superior qualities via hybridization compared to natural composites in fibers. In general, natural fiber compounds have lower characteristics, limiting the use of natural composites reinforced by fiber. Significant effort was spent in enhancing the mechanical characteristics of this group of materials to increase their strengths and applications, especially via the hybridization process, by manipulating the characteristics of fiber-reinforced composite materials. Current studies concentrate on enhancing the understanding of natural fiber-matrix adhesion, enhancing processing methods, and natural fiber compatibility. The optimal and resilient conceptions have also been addressed due to the inherently more significant variabilities. Moreover, much research has tackled natural fiber reinforced hybrid composite costs. In addition, this review article aims to offer a review of the variables that lead to the mechanical and structural failure of natural fiber reinforced polymer composites, as well as an overview of the details and costings of the composites.

## 1. Introduction

Composite materials are produced from a combination of two or more elements that are easily distinguishable to enhance the characteristics of the individual element [1,2]. Newly invented materials may be favored for various reasons, e.g., they are stronger, more lightweight, and less costly than existing materials [3,4,5]. In general, the individual materials making up composites are known as constituents. Principally, most composites comprise two constituent materials—reinforcement and matrix—however, in general, the composites may contain not only two components, matrix and reinforcement, but also other types of components: fillers, compatibilizers, coupling agents, pigments, lubricants, surfactants, solvents, etc. Only the simplest textile-based composites, also called textolites, contain two constituents, polymer matrix and reinforcement such as natural, synthetic or hybrid fibers, or fabrics. The reinforcement is significantly stiffer and stronger than the matrix, contributing to the composites’ superior characteristics [6,7]. The main functions of the polymer matrix in textile-based composites are to bind reinforcements (fabric, fibers, or nanofibers) and maintain the integrity of the composite.

**Figure 1:**
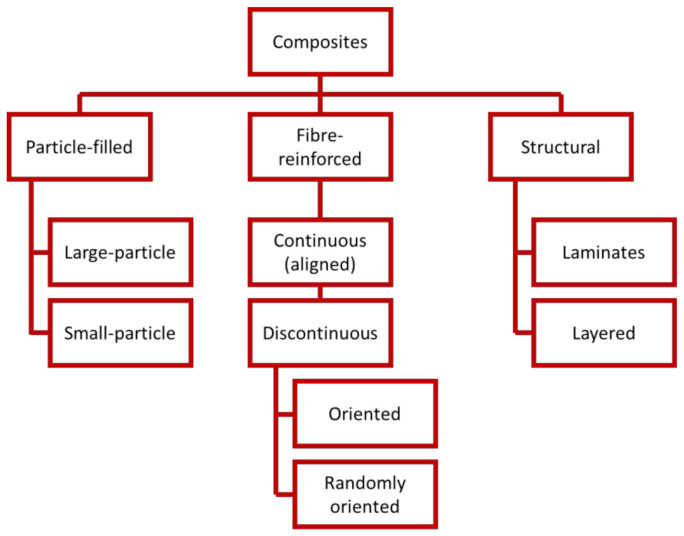
Types of reinforcements in composites. Redrawn with permission from Saba and Jawaid [12].

Composites’ reinforcements can be fabrics elements, fibers, or nanofibers [8,9,10]. Fiber is defined as one very long axis with two other axes that frequently are either circular or near-circular. The fibers have a pronounced axial orientation. As is known, Young’s modulus and tensile strength of fiber in the longitudinal direction (along the fiber axis) is usually an order of magnitude higher than in the lateral direction of the fiber. Nanofibers have an ideal form; however, they are smaller in diameter and length compared to fibers [11]. Figure 1 shows the type of reinforcements in composites.

The matrix might be thermoset, thermoplastic, or biopolymer. Polyvinyl chloride (PVC) is the most common thermoplastic matrix used in natural fiber composites. Besides this, due to the limited compatibility of nonpolar hydrophobic polyethylene and polypropylene with polar and hydrophilic natural cellulose fibers, these thermoplastics typically are not employed as a matrix for natural cellulose fibers. Meanwhile, polyester, epoxy, and phenolics are the most often used thermoset polymers [13,14].

Nowadays, the availability of bio-based polymer matrices on the market is comparatively meager; however, it has been speedily expanding, thanks to a huge number of industrial investigations and continuous research into the advantages of these materials, as well as their practicability in actual applications. The preliminary data on the essential characteristics of composites attained from eco-based matrices were reported in [15,16].

## 2. Natural Fiber (NF)

**Figure 2:**
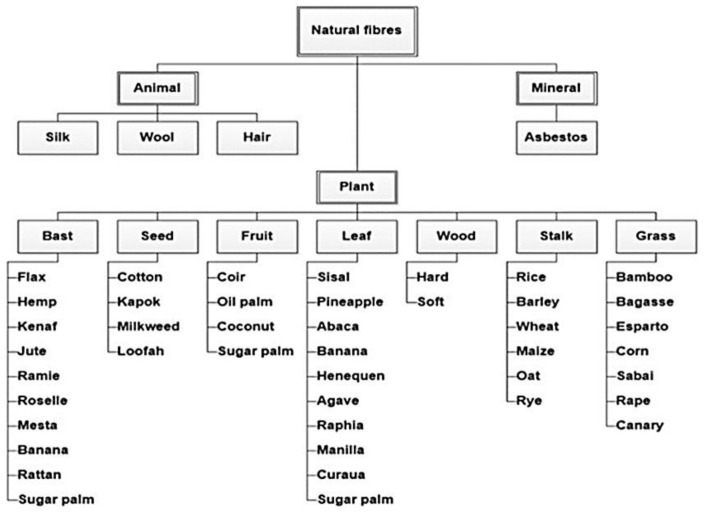
Classifications of natural fibers. Redrawn with permission from Jawaid and Khalil [12].

Fiber is the continuous filaments hair-like material of elongated piece that is similar to a thread, while fibers is a group of fiber that can be coiled into rope, filaments, or thread [17,18,19]. They are useful as of the composite materials’ element and were also formed into sheets to produce felt or paper. Fibers are categorized into three groups: (1) natural fiber and (2) man-made, and (3) synthetic fiber. Natural fibers are either animal, plant or mineral-based that are extracted from nature without compromising the environment. Commonly used animal-based natural fibers including feather, wool, silk, hair, etc. Examples of plant-based natural fibers are banana, jute, hemp, bamboo, flax, sisal, etc., which are broadly applied to manufacture natural fiber reinforced polymer (NFRP) composites [20]. The classifications of natural fibers are shown in Figure 2, and the annual natural fiber production is presented in Table 1.

Over the past few years, natural fibers have become eminent reinforcing fibers in polymer-matrix composites (PMC). They offer rapidly increasing and abundant characteristics, allowing them to be obtained at a small cost. Numerous attempts in terms of studies have been performed worldwide to prove the eligibility of natural fiber-based composites to substitute the synthetic as newly engineered fibers. Due to the growing need for renewable, cost-effective, and environmentally friendly materials, the use of natural fibers as composite materials’ reinforcement has proliferated over the years.

When compared to glass fiber composites, natural fiber composites are more advantageous for being more lightweight, biodegradable in nature, ease of machinability, zero toxicity, cheap cost, convenience, and non-abrasive nature [13,21,22]. Many natural fiber composites are reputable and have been satisfactorily proven in research. The number of natural fiber composites has been investigated previously in terms of their physical and mechanical properties, such as arrowroot, hemp, sisal, coir, jute, kenaf, date, pine cone, vakka and bamboo [23,24,25,26,27].

**Table 1:** Annual natural fibers production. Extracted with permission from [28].

Natural Fiber	Source	World Production $\left(\times 10^{3}\;\mathrm{Tons}\right)$
Abaca	Leaf	70
Banana	Stem	200
Bamboo	Stem	10,000
Broom	Stem	Abundant
Coir	Fruit	100
Cotton lint	Seed	18,500
Elephant	Stem	Abundant
Flax	Stem	810
Hemp	Stem	215
Jute	Stem	2500
Kenaf	Stem	770
Linseed	Fruit	Abundant
Oil Palm Fruit	Fruit	Abundant
Ramie	Stem	100
Rice Husk	Grain	Abundant
Roselle	Stem	250
Sisal	Leaf	380
Sun hemp	Stem	70
Wood	Stem	1,750,000

## 3. Recent Development of Natural Fiber Reinforced Hybrid Composites

It is known that natural fibers possess some limitations compared with those common fibers such as glass and carbon, where it is having more inferior mechanical properties and a higher water absorption [29]. Therefore, an introduction of hybrid biocomposites consists of two or more fibers in one matrix is seen as a solution to enhance the natural fiber-reinforced polymer composites’ properties. Zahra et al. [30] stated that hybridizing one natural fiber with another natural fiber/synthetic fiber in one matrix will improve it’s thermal and mechanical than the individual fiber composites [31]. This has shown that hybrid composites are more reliable for various applications, besides being more environmentally friendly.

The hybridization of natural fiber-based reinforced polymer composites can be done through a combination of natural–natural fibers, natural–synthetic fibers, natural fiber with carbonaceous materials, and natural fiber with metal [32]. Due to their varied properties and considerations of interfacial adhesion, hybrid natural fiber composite materials are facing difficulties in fabrication. Composites are manufactured in a variety of ways, such as through basic mixing and open or closed molding. Many factors can affect the interactions between the fiber and matrix, for example, and could be mild owing to the existing van der Waals forces, hydrogen bonding, and weak electrostatic interactions. In addition, a good interaction could also exist due to the chemical interactions between those materials. Therefore, studies on hybrid natural fiber composites keep increasing in order to discover the ability of hybrids to be a possible alternative, replacing various petroleum-based products. Some examples of the studies of hybrid natural fiber composites are presented in Table 2.

### 3.1. Sugar Palm Fiber Reinforced Hybrid Composites

**Figure 3:**
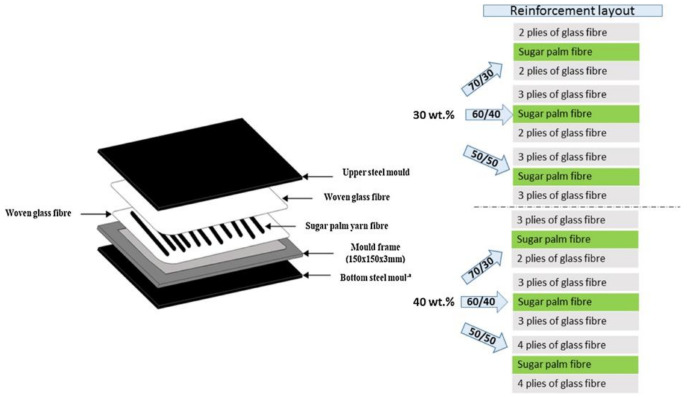
Schematic diagram of layup segmentation and reinforcement layout [139]. Extracted with permission from Elsevier.

*Arenga Pinnata* (also known as sugar palm) is a versatile palm species with wide applications in foods and beverages [133], timber commodities [134], biofibers [135,136,137,138,139,140,141], biopolymers [142,143] and biocomposites [144,145,146,147,148,149,150,151,152,153]. Sugar palm fibers are recognized for their great durability, as well as their resistance to seawater. Sugar palm fibers have been used to produce ropes for ship cordages that have confirmed the good performance in saltwater [154]. Via the hand-lay-up technique, Misri et al. [28] manufactured a small boat using innovative material, a hybrid of sugar palm fiber and fiberglass-reinforced unsaturated polyester. The mechanical properties of the hybrid boat were investigated via the tensile and impact tests and were found the increased impact strength of 2.471 kJ/m^2^ and tensile modulus of 1840.6 MPa. Sanyang et al. [155] reported that the sugar palm fiber demonstrated a lower density than the commercial E-glass fiber of 1.22–1.26 kg/m^3^ and 2.55 kg/m^3^, respectively. This consequently resulted in the weight reduction of the manufactured boat by 50%. Recently, sugar palm fiber has been investigated as a hybrid reinforcement [154,156,157,158,159]. Certain precautions must be considered in the development of these novel natural fiber composites in terms of applicability. For instance, critical assessment and characterization of these composites for practical use in more comprehensive applications. Figure 3 presents the schematic diagram of layout segmentation and reinforcement layout of sugar palm/glass fiber designed by Nurazzi et al. [139] The results revealed improvements in thermal stability, char residue, as well as decomposition temperature as the glass fibers and sugar palm ratios, were raised to 50/50 for both 30 wt.% and 40 wt.% of fiber loadings.

Afzaluddin et al. [160] investigated the influence of the different treatments with 2% silane (TSSP), 6% alkaline (TNSP), and a combination of 6% alkaline–2% silane (TNSSP) on the thermal and physical characteristics of sugar palm/glass/thermoplastic polyurethane hybrid composites. The findings showed that the combined alkaline–silane-treated hybrid composites (TNSSP) displayed the minimum water absorption, thickness swelling, and density as with other hybrid composites. Besides this, good thermal stability was observed in the treated sugar palm fiber-based composites compared to the untreated ones. It is suggested that treated sugar palm/glass/thermoplastic polyurethane hybrid composites can fit automotive component applications. The results of this research are aligned with other studies conducted on the treated sugar palm fiber-reinforced polymer hybrid composites [33,35,36,161].

### 3.2. Kenaf Fiber Reinforced Hybrid Composites

Kenaf (*Hibiscus cannabinus* L.) is among the most common natural fibers used as polymer matrix composite (PMC) reinforcement. It is an annual herbaceous plant that can be cultivated in a variety of climates and grows to more than 3 m in 3 months, even in temperate climates [162]. Davoodi et al. [48] replaced an automobile bumper beam with a hybrid kenaf/glass-reinforced epoxy composite to reduce environmental impact while maintaining the requisite strength.

**Figure 4:**
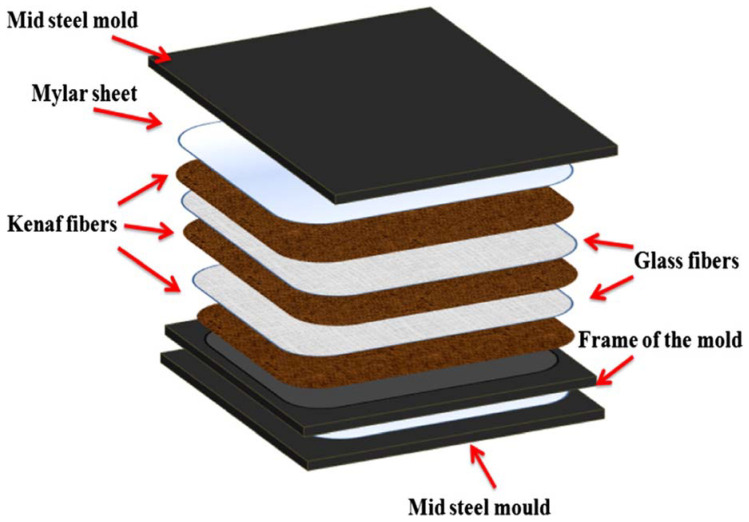
The sequence of kenaf and glass fibers and matrix in between mold for hybridization conducted by Atiqah et al. [48]. Extracted with permission from Elsevier.

The development of kenaf-glass (KG) fiber reinforced unsaturated polyester (UPE) hybrid composite was performed by Atiqah et al. [49] via the process of sheet molding compound for structural applications. The ratio of 70:30 (by volume) of UPE and KG fibers in a mat form is used using untreated and treated kenaf fiber. During the mercerisation process, the kenaf fiber was alkaline treated for 3 h using a 6% sodium hydroxide (NaOH) diluted solution. Figure 4 shows the sequence of kenaf and glass fibers and matrix in between a mild steel mold for the fabrication of a hybrid composite. The result demonstrated that the highest tensile, flexural and impact strengths were attained from the treated kenaf containing 15/15 *v*/*v* KG fibers reinforced UPE hybrid composite. Besides this, the main fracture mode of composites observed under the scanning electron microscopy fractography was fiber debonding, cracking, and pull-out. Better interfacial bonding between the matrix was found in the kenaf treated 15/15 *v*/*v* KG reinforced hybrid composite than with other combinations. The hybridization of natural fibers, particularly synthetic and kenaf fibers, is an excellent method to improve the mechanical characteristics of the fabricated hybrid composite, as reported in many works [51,52,53,54,55,56,57,58,59,163].

The fabrication of kenaf fiber reinforced polypropylene (PP) sheets into a sheet form have been successfully carried out via thermoforming, where the optimum process is the compression molding that employs a layered sifting of a micro-fine PP powder and chopped kenaf fibers [164]. 30 and 40 wt.% fiber contents provide sufficient reinforcement which improves the PP matrix’s strength. The strength of the molded kenaf–PP composites was evidenced to possess better flexural and tensile strengths compared to the strength of other molded natural fiber composites, e.g., coir, kenaf, and sisal reinforced thermoplastics. The economic advantage of using kenaf composites over E-glass and other natural fibers is the possibility to analyze the elastic modulus data. The fabricated kenaf maleated PP composites exhibited a greater modulus/cost and an advanced specific modulus compared to coir, sisal, and E-glass. Therefore, they deliver a choice for substituting the currently used materials with a lower-cost alternative with a higher strength that is also environmentally friendly.

The wood flour/kenaf fiber and PP hybrid composites were set to evaluate the hybrid outcome on the properties of the composites [67]. The findings demonstrated that non-hybrid composites (wood flour and kenaf fiber) revealed the lowest moduli compared with the hybrid composites; in addition, moduli of the hybrid composites strictly adhered with the relationship between the fiber reinforcement to wood filler. It was more likely to estimate the elastic modulus of composites using the hybrid mixtures equation rather than with the Halpin–Tsai equation. The influence of natural rubber toughening with polyester resin as the matrix on kenaf fibers were also studied by Bonnia et al. [165].

### 3.3. Oil Palm Fiber Reinforced Hybrid Composites (OPRPC)

Oil palm, *Elaeis guineensis* consist of two *Arecaceae* or palm family species. Oil palm empty fruit bunch fibers are among potential reinforcement fibers for polymer composites [166,167]. Agarwal et al. [167] examined the stress relaxation behavior in phenol-formaldehyde resin reinforced with oil palm empty fruit bunch fibers and hybrid composites composed of oil palm fibers and glass fibers. The examination of the influences of fiber treatment, loading, strain level, and physical aging on the stress relaxation behavior and the calculation of the rate of relaxation at different time intervals were performed to describe the progressive alterations in the relaxation mechanisms [168].

**Figure 5:**
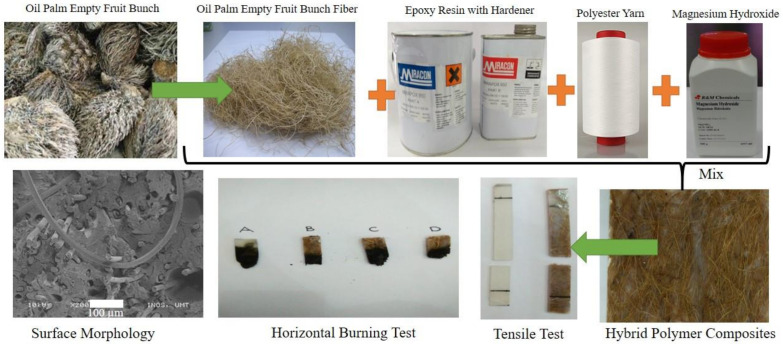
Extraction of OPEBF, and fabrication of OPEBF/polyester yarn, magnesium hydroxide reinforced epoxy resin hybrid composite. Extracted from [69] with permission.

Suriani et al. [69] introduced the oil palm empty fruit bunch (OPEFB) fiber and Mg(OH)_2_ into epoxy resin to obtain a hybrid composite, as shown in Figure 5. Four specimens were considered; (1) specimen A (blank, 0% fiber), (2) specimen B (20% fiber), (3) specimen C (35% fiber), and (4) specimen D (50% fiber). The used reinforcing and fire retarding additives were the PET yarn and magnesium hydroxide, respectively. The burning test result exhibited better flammability in specimen B, with the lowest average burning rate of 11.47 mm/min. Specimen A demonstrated the highest tensile strength of 10.79 N/mm^2^. An SEM morphological test revealed rising surface defects by the rupture that resulted in the decline of the composites’ tensile properties. The authors summarized that the tensile properties and flammability of OPEFB fiber-reinforced fire-retardant epoxy composites weakened with the increments in the fiber volume content at the optimum 20% loading of 11.47 mm/min and 4.29 kPa, respectively. Another study conducted by Farah et al. [70] for the characterization of hybrid epoxy composites containing oil palm empty fruit bunch/woven kenaf fabric reinforcement demonstrated that the increased oil palm fiber content leads to an increase in the impact strength of the hybrid composite. It is described by the other circumstance in which randomly oriented empty fiber bunches (EFB) has a moderate interfacial interaction with epoxy that is vital to attaining a higher impact strength. An investigation into the impact of oil extraction, compounding processes and fiber loading [76], as well as matrix alteration on the mechanical characteristics of oil palm empty fruit bunch filled PP composites was also conducted [71]. Moreover, oil palm empty fruit bunch fiber/PP composites and oil palm-originated cellulose/PP composites were compared [169].

The effect of chemical alteration of the composites containing oil palm/phenol formaldehyde was studied by comparing polyester and epoxy matrices. In addition, the dielectric relaxation and the fiber orientation effect on the dynamic electrical properties of palm tree fiber-reinforced polyester composites were studied [69,170,171,172].

### 3.4. Pineapple Leaf Fiber Reinforced Hybrid Composites (PARPC)

Pineapple—*Ananas comosus*—is a tropical plant native to Brazil, with long leaves containing fibers that have a high cellulose content. They are cheap and easily available. In addition, pineapple leaves possess the possibility to be used as a reinforcing agent in polymers. At present, pineapple leaf fibers are the by-products of pineapple farming, making these inexpensive fibers accessible for industrial use, especially for the reinforcement of polycarbonate to manufacture composites [173,174]. The composite fabricated from silane-treated pineapple leaf fibers revealed the most excellent impact and tensile strengths. Thermogravimetric analysis data demonstrated that the composites’ thermal stability was poorer than neat polycarbonate resin, which also declined with the rising content of pineapple leaf fiber. The Transient Plane Source (TPS) technique was employed to study the thermal conductivity and diffusivity of phenol-formaldehyde composites reinforced with pineapple leaf fibers [175]. The composites’ effective thermal diffusivity and conductivity were found to decrease compared to pure phenol-formaldehyde due to the increment in the fiber loading fraction.

Various efforts to improve pineapple leaf fiber’s quality have been carried out via several surface alterations, e.g., alkali treatment, dewaxing, cyanoethylation, and grafting acrylonitrile onto dewaxed fibers [176]. The mechanical characteristics were optimum at 30 wt.% fiber loading. From all surface modifications, 10% acrylonitrile grafted fiber-reinforced polyester composite exhibited a maximum tensile strength of 48.36 MPa. However, cyano-ethylated fiber composites demonstrated a better impact and flexural strengths of 27% and 41% more, respectively, compared to unmodified composite. The effect fiber content and surface treatment were also studied using natural rubber and PP as the matrices [177,178].

Hashim et al. [77] conducted a study using a vacuum infusion technique on the influence of stacking sequence and ply orientation on the mechanical characteristics of pineapple leaf fiber (PALF)/Carbon hybrid laminate composites. The tensile and flexural tests’ findings displayed that the laminate with inner carbon plies and ply orientation [0°, 90°] resulted in the maximum tensile strength as well as modulus of 187.67 MPa and 5.23 GPa, respectively. Fracture properties of the composite laminates were investigated using scanning electron microscopy and it was discovered that the failure was started at the weakest fiber layer. This phenomenon might be due to the failure modes, including delamination, debonding, matrix crack, fiber breaking, and fiber pull-out [179,180,181,182,183,184,185,186].

**Figure 6:**
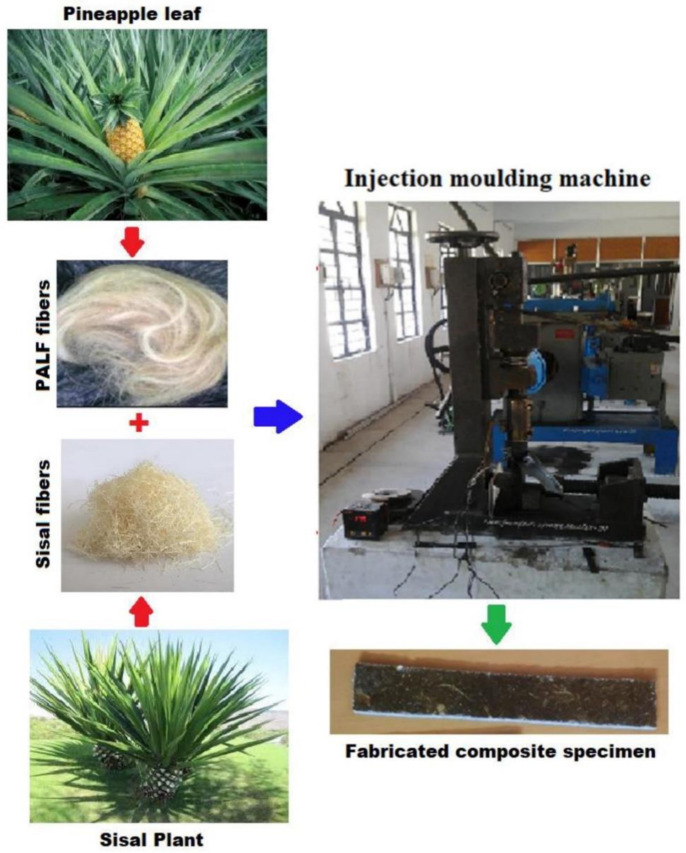
The fabrication process of pineapple leaf (PALF) and sisal fiber reinforced polyester composites using injection molding technique. Extracted from ref. [82] with permission.

In a work conducted by Sathees Kumar et al. [82], the effects of fiber loading on the mechanical characteristics of reinforced polyester reinforced with sisal and pineapple leaf (PALF) fibers using an injection molding technique were studied, as shown in Figure 6. Figure 6 showed that equal weight % share of PALF and sisal enhanced the overall mechanical attributes, e.g., ductile strength (207 MPa), bending strength (90.3 MPa), impact (29 J/m^2^), and hardness (83.7). The mechanical test results revealed a regular trend of an increase in flexural, tensile, impact, and hardness with the addition of PALF fibers, and this was supported by various works [80,81,187,188]. Besides that, they concluded that this type of composite material could be valuable for multiple industries, including automotive and construction fields.

### 3.5. Bamboo Fiber Reinforced Hybrid Composites (BRPC)

**Figure 7:**
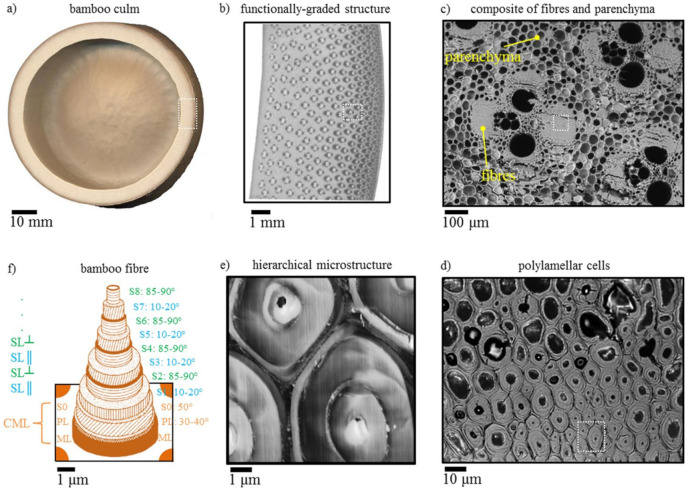
(**a**) Bamboo culm, (**b**) bamboo culm cross-section, (**c**) vascular bundle, (**d**) polylamellar cells, (**e**) microstructure fibers, and (**f**) bamboo’s model of polylamellae structure. Extracted with permission from Ref. [189].

Bamboo (*Bambusa* Shreb.) is a perennial plant that is able to reach a height of 40 m in monsoon climates. Figure 7 displays the morphological structure of the bamboo fiber [189]. Bamboo is used in carpentry, construction, plaiting, and weaving. Curtains are made from bamboo fiber and absorb various wavelengths of ultraviolet radiation, resulting in less harmful radiation to the human body.

Osorio et al. [83] developed a novel mechanical extraction process of long bamboo fibers (*Guadua angustifolia*) for use as a reinforcing agent in structural composites. The effectiveness of the new reinforcement was evaluated by fabricating the composites containing unidirectional bamboo fiber/epoxy (BFC) with alkali-treated and untreated fibers. Two orientations of fiber (transverse and longitudinal) were employed in the flexural tests. When untreated fibers were utilized, the composite’s longitudinal flexural strength was greater, whereas treatment increased the longitudinal flexural stiffness. For untreated bamboo in epoxy, the transverse strength rose with the decreasing alkali concentrations, while its three-point bending strength was already extremely high at approximately 33 MPa. They concluded that bamboo fiber offers a natural and renewable alternative to glass fiber and is helpful as traditional natural fiber reinforcement in a variety of applications where glass fiber and conventional natural fibers are already in use.

When preparing bamboo fiber-reinforced composites, characteristics of material and method affect the produced bamboo hybrid composite’s quality [190]. A novel composite material fabricated from a right reinforcement material and the matrix combination is able to fulfill a specific application’s requirements [191]. The benefits offered by composite materials include their excellent strength, their lightweight, and their moldability. In contrast, polymeric fiber composites have a high raw material cost. Numerous methods of fabrication have been developed to manufacture bamboo-reinforced plastics as well as hybrid composites, e.g., cold and hot presses, and injection molding. These procedures have been used on various bamboo-reinforced polymeric materials to make hybrid composites [84,85,86,87,192].

Bamboo fiber, aliphatic polyester, and polyolefin blends are particularly appealing. Blending bamboo fiber with polypropylene (PP) and polylactic acid (PLA) will lead to enhanced chemical, mechanical and thermal characteristics. The materials that result may be turned into products with more convenience and at a lower cost. The development of novel composites using a polypropylene (PP)/polylactic acid (PLA) matrix and filler bamboo fiber (BF) results in modifications in the raw thermoplastic’s processability, morphology, and rheological characteristics [193]. Maleic anhydride grafted polypropylene (MAH-g-PP) was used at the filler–matrix interface to increase PP, PLA, and BF interface strength and to improve PLA dispersion and composite toughness. The addition of MAH-g-PP to composites resulted in positive morphological and rheological alterations, which were linked to enhanced PLA dispersion and increased bamboo fiber–matrix interactions.

Glass and bamboo fibers were used to create hybrid composites made of isophthalate polyester and vinyl ester resin. The optimized glass fiber reinforced composites were submitted to dynamical mechanical analysis to evaluate the dynamic characteristics as a function of temperature and frequency with 25, 50, and 75% of glass fibers substituted by bamboo fibers. The storage modulus E′ was spotted to drop as the wt.% of bamboo fibers increased. The loss modulus was observed to reduce with loading; however, the damping property increased significantly. Fiber–matrix bonding was visible in scanning electron micrographs of composite flexural fracture surfaces.

### 3.6. Jute Fiber Reinforced Hybrid Composites (JRPC)

**Figure 8:**
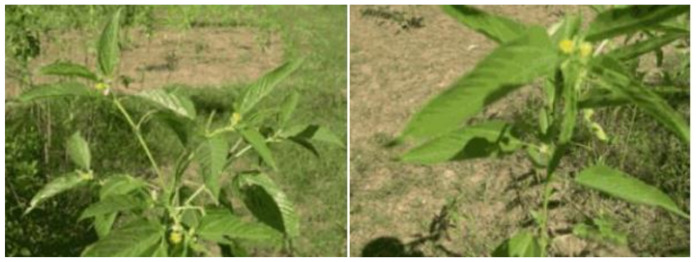
Jute plant. Reproduced with permission from Ramesh et al. [195].

**Figure 9:**
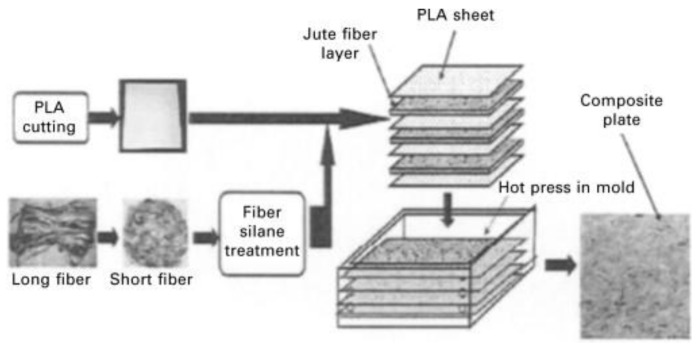
Fabrication procedure of jute fiber reinforced polymer composites [196]. Reproduced with permission.

Jute is obtained from *Corchorus* genus plants that have about 100 species. At present, jute dominates the highest production volume among bast fibers and is globally available as one of the cheapest natural fibers. Jute is best grown in India, Bangladesh, and China. Figure 8 shows that jute plants are being cultivated for fiber production. Sarkar and Ray [94] studied the alkali-treated jute fiber reinforced with vinyl ester resin using the compression molding technique, as shown in Figure 9. The mechanical, dynamic, thermal, and impact fatigue behavior were compared with the untreated jute fiber–vinyl ester composites. Better fiber dispersion resulted from an extended alkali treatment that eliminated hemicelluloses, hence improving the crystallinity. All properties of mechanical, dynamic, thermal and impact were excellent due to the longer treatment period, concentration, and conditions during the alkali treatment [94].

Jute fiber reinforced hybrid composites have a number of advantages, e.g., a low specific gravity, increased tensile and compressive strength and modulus, and improved fatigue strength [194]. In work conducted by Prasath et al. [102], polyester-based polymer composites were developed by a compression molding technique with different stacking sequences of basalt and jute fabrics into the general-purpose polyester matrix. The result showed that a combination of pure basalt fiber maintained higher values during flexural and tensile tests. However, in the impact test, basalt fiber was somewhat lesser than jute fiber-reinforced composites [102].

Ramana and Ramprasad [95] conducted a study on a new hybrid composite developed from jute and carbon fiber reinforced epoxy composite and discussed its superiority or inferiority compared to jute-epoxy and carbon-epoxy composites so that the extent of the utility of the newly developed composite could be established. The hand layup technique was utilized for the composite preparation, and the total fiber content considered was 45%. The newly developed composites, for instance, jute and carbon-epoxy hybrid composites, can replace carbon-epoxy composites without much loss of tensile and flexural strengths as well as a flexural modulus and with improved ductility and impact strength [95].

Mohanty et al. [197] studied the surface modification influence on the biodegradability and mechanical properties of jute/Biopol and jute/PA (Poly Amide) composites. More than 50%, 30%, and 90% in tensile, bending, and impact strengths were found and compared to the values obtained for pure Biopol sheets. In addition, greater than 50% weight loss was observed after 150 days of compost burial of the jute/Biopol composites. The hybridization effects on tensile characteristics of jute–cotton woven fabric reinforced polyester composites were investigated as functions of fiber orientation, content, and texture of roving. Tensile characteristics along the alignment direction of jute roving (transverse to cotton roving alignment) rose continuously with fiber content until 50% before showing a tendency to decline. The composites’ tensile strength value at 50% fiber content parallel to the jute roving was approximately 220% greater than pure polyester resin [101].

The evaluations conducted on jute fiber reinforced PP composites include a matrix modification effect, gamma radiation influence, interfacial adhesion effect on creep and dynamic mechanical behaviors, silane coupling agent influence, and natural rubber effect [198,199,200,201]. The jute/plastic composites properties were studied, comprising crystallinity, thermal stability, transesterification, modification, durability, weathering, eco-design of automotive components, fiber orientation on frictional and wear behaviors, and alkylation [198,202,203,204,205,206,207]. Jute fiber reinforced composites used polyester resin matrix, and examinations were carried out on the water absorption and dielectric behavior relationship [208], properties of elasticity, fracture criteria and notched strength [96], characterization of impact damage [209], thermal behavior and weathering [210], and silane treatment effect [211].

### 3.7. Hemp Fiber Reinforced Hybrid Composites (HRPC)

Hemp is another renowned bast fiber crop, an annual plant in the Cannabis family that cultivates in temperate climates. As a European Union subsidy for non-food agriculture, many current initiatives are progressing for its development in Europe. PP composites with hemp fibers were functionalized by the reactions of melt grafting using glycidyl methacrylate (GMA) and were manufactured via batch mixing [212]. The fibers and PP matrix modifications and various compatibilizer additions were conducted to enhance the interactions of the fiber–matrix. In comparison with the unaltered composite, chemical bonding between the fiber and the polymer (PP/Hemp) resulted in improved fiber distribution in the PP matrix as well as higher interfacial adhesion in the modified composite. Matrix and fiber modifications highly influenced the phase behavior and thermal stability of the composites. The alterations in the crystallization behavior and spherulitic morphology of PP in the composites were analyzed due to the hemp fibers’ nucleating effect. Additionally, with increasing modified hemp content, a significant rise in the PP isothermal crystallization rate (120–138 °C) was observed. All composites demonstrated a higher tensile modulus (about 2.9 GPa) and lower elongation at break when compared to plain PP. Still, compatibilization with modified PP (10 phr) boosted the stiffness of the composites due to better fiber–matrix interfacial adhesion.

**Figure 10:**
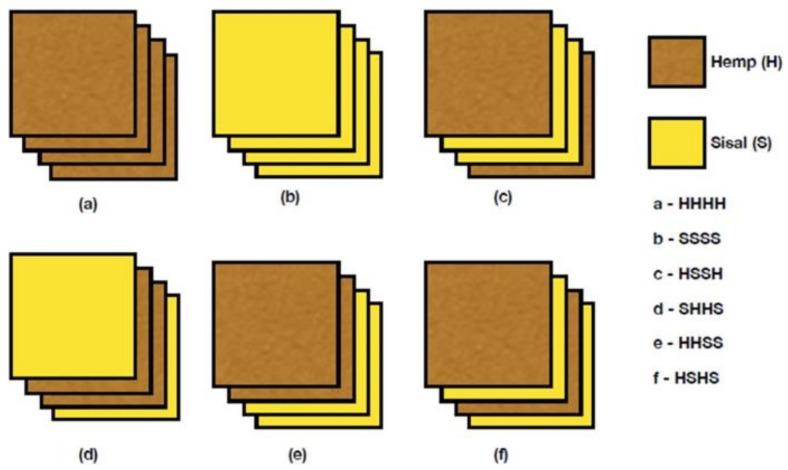
Different layering arrangements of sisal and hemp fiber mats [104]. Reproduced with permission.

Ramesh et al. [103] fabricated hybrid composites using carbon, alkaline-treated, and untreated hemp fibers and investigated their properties. The hybrid composites possessed maximum tensile, flexural, impact, and shear strengths of 61.4 MPa, 122.4 MPa, 4.2 J/mm^2^, and 25.5 MPa, respectively. In addition, from the composites’ mechanical properties, the alkaline-treated composites exhibited better performance [103]. Thiagamani [104] fabricated hybrid bio-composites using the green epoxy matrix, reinforced with sisal (S) and hemp (H) fiber mats via the cost-effective hand lay-up method and hot press employing different stacking sequences, as presented in Figure 10. As the stacking sequence was changed, the tensile strength varied slightly, where the intercalated arrangement (HSHS) hybrid composite demonstrated a maximum tensile modulus compared with the other hybrid counterparts. Hybrid composites (SHHS and HSSH) possessed a compressive strength that was 40% more than the other layering configurations, and the HHHH sample had the maximum ILSS of 4.08 MPa [104].

Li and co-workers [213,214,215] investigated the effects of chelators, white rot fungi, and enzyme treatments towards hemp fiber separation from bundles and enhanced the hemp fibers’ interfacial interaction with the PP matrix. The findings indicated that treated fiber composites had a greater interfacial shear strength than untreated fiber composites, a conclusion that was corroborated by a large body of literature [92,174,216,217,218,219]. This demonstrates that the white rot fungal treatment increased the interfacial attachment of hemp fiber to PP. Composites made of chelator concentrate treated hemp fibers exhibited the maximum tensile strength, measuring 42 MPa, a 19% improvement above composites made of untreated hemp fiber. Additionally, hemp fiber reinforced PP composites showed fascinating recyclability [220]. The findings demonstrated that despite the high number of reprocessing cycles, the mechanical properties of hemp fiber/PP composites were well maintained. Newtonian viscosity reduced as the number of cycles increased, indicating a decline in chain scissions and molecular weight caused by reprocessing. Another possible explanation for the decrease in viscosity was the shortening of the fibers during reprocessing.

In addition, several investigations on the hemp-based composites have also been conducted in terms of their effect on the falling weight impact properties [221], composites’ properties and performances for curved pipes [222], impact load performance of resin transfer molded composites [223], composites’ micromechanics [224], the influence of soybean oil and nano clay hybrid blends [105], as well as the practicality of untreated hemp as the biocomposites’ fiber source [225]. Kunanopparat et al. [226] investigated the viability of using wheat gluten as a hemp fiber-reinforced composite matrix, focusing on the effect of thermal treatment and plasticization on mechanical properties.

### 3.8. Flax Fiber Reinforced Hybrid Composites (FRPC)

Flax is among the world’s oldest fiber crops, containing bast fiber that is cultivated in temperate regions. Flax bast fiber is often utilized for applications in the higher value-added textile industries. Recently, flax has been broadly used in composites. The dynamic and static mechanical characteristics of nonwoven-based flax fiber reinforced PP composites were investigated while taking into account the effect of zein coupling agent, a zein protein [227]. It was discovered that composites containing zein protein as a coupling agent have improved mechanical properties. The composites’ storage modulus increased with the addition of a zein coupling agent due to the increased interfacial adhesion. The diameter and position of flax fibers in the stems are used to evaluate their tensile mechanical properties. The substantial dispersion of these attributes is a result of the fiber’s longitudinal axis size variation. The increased mechanical qualities of the fibers originating from the stem’s center are related to their cell walls’ chemical composition. The mechanical characteristics of unidirectional flax fiber/epoxy matrix composites were investigated in terms of their fiber content. The composites’ properties were poorer than predicted from the characteristics of a single fiber.

**Figure 11:**
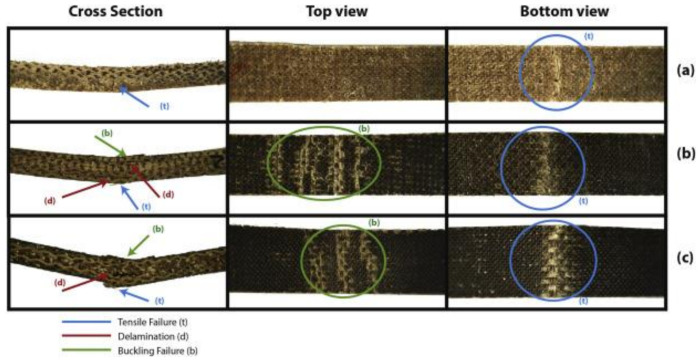
Jute-basalt reinforced hybrid composites including (**a**) jute fibre, (**b**) intercalate and (**c**) sandwich laminates [228]. Reproduced with permission, Elsevier.

Chaudhary et al. [100] developed and characterized the composites made from natural fibers (hemp/epoxy, jute/epoxy, flax/epoxy) and their hybrid composites (hemp/flax/epoxy, jute/hemp/epoxy, and jute/hemp/flax/epoxy). Among hemp/epoxy, jute/epoxy, and flax/epoxy, a higher hardness (98 Shore-D) and tensile strength (46.2 MPa) was shown by flax/epoxy composite. In contrast, better impact and flexural strengths were exhibited by jute/epoxy (7.68 kJ/m^2^) and hemp/epoxy (85.59 MPa) composites, respectively. In general, hybrid composites exhibited better mechanical performance. For example, jute/hemp/flax/epoxy hybrid composite demonstrated the highest tensile modulus strength and sn impact strength of 1.88 GPa, 58.59 MPa, and 10.19, kJ/m^2^, respectively. In contrast, the flexural strength of jute/hemp/epoxy hybrid composite was maximum, 86.6 MPa [100]. A similar trend has been shown by Fiore et al. [228], fabricating jute-basalt reinforced hybrid composites via the hand-lay-up method, as presented in Figure 11, for structural applications.

Paturel and Dhakal [109] studied the moisture absorption influence on flax and flax/glass hybrid laminates to investigate their low-velocity impact behavior. Three different composite laminates, (1) flax fiber reinforced vinyl ester, (2) flax fiber hybridized glass fiber, and (3) glass fiber reinforced vinyl ester, were manufactured via the resin infusion method. Moisture immersion tests were conducted by immersing various specimens in seawater baths at room temperature and 70 °C at various periods of time. The low velocity falling weight impact test was conducted at a 25 J incident energy level, and the impact damage behavior was analyzed using scanning electron microscopy (SEM) and X-ray microcomputed tomography (micro CT) under both aging circumstances. With glass fiber hybridization, the percentage of moisture taken in by flax vinyl ester specimens was lowered. The maximum weight growth percentages for flax fiber, flax/glass hybrid, and glass fiber reinforced composites immersed in water at room temperature for 696 h were 3.97%, 1.93%, and 0.431%, respectively. When compared to a flax/vinyl ester composite without hybridization, the hybrid composite demonstrated increased load and energy, demonstrating that the hybrid system is a viable technique for improving the structural performance of natural fiber composites. At room temperature, the composites’ moisture absorption behavior was found to follow Fickian behavior [109].

Numerous studies on the composites of flax fiber/polypropylene have been conducted. However, these researches concentrated on various variables, natural fiber thermoplastic mat (NMT) and glass fiber thermoplastic mat (GMT) comparison [229], the effect of glass fiber hybridization and fiber/matrix modification [230], the influence of fiber treatment on crystallization and thermal properties [110], surface treatment influence on the interface by thermoplastic starch, glycerol triacetate, boiled flax yarn, and -methacryl oxypropyl trimethoxy-silane [231], matrices comparison (PP and PLA) on the properties of composites [111], material and processing parameters effects [232], and processing methods influence [233]. Buttlar [234] reported the viability of flax fiber composite applications in the bus and automotive industries.

The bio-technical fiber modification effects are ascribed with: (i) toughness and fracture behavior, (ii) alkaline fiber treatment influence on unidirectional composites, and (iii) processing parameter influence on the successive flax fiber’s decortication steps (retting, scotching, and hackling) towards the flax fiber reinforced epoxy composites [112,235,236,237]. Thermal degradation and fire resistance of flax fiber composites reinforced with polyester resin were studied, as well as the influence of chemical treatments on surface properties and adhesion, and also the influence of chemical treatments on the water absorption and mechanical characteristics [238,239]. Three soybean oil-based resins, methacrylic anhydride modified soybean oil, methacrylated soybean oil, and acetic anhydride modified soybean oil, were also used as matrices for the flax fiber-reinforced biocomposites.

### 3.9. Ramie Fiber Reinforced Hybrid Composites (RRPC)

**Figure 12:**
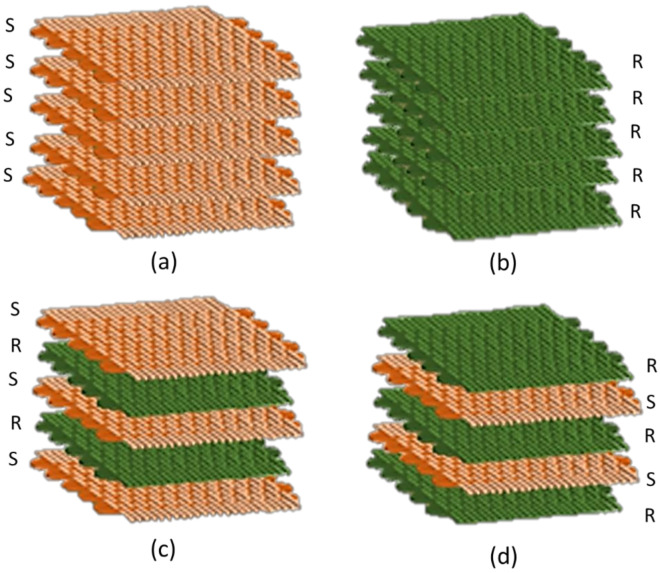
Various hybrid composite stacking sequences: (**a**) SSSSS, (**b**) RRRRR, (**c**) SRSRS, and (**d**) RSRSR [45]. Extracted from Ref. [45] with permission.

Ramie is a plant from the Urticaceae (*Boehmeria* spp.) family that comprises approximately 100 species. The exploitation of ramie is for use as textile fiber with two limiting factors: production regions as well as a need for more considerable pre-treatment than other commercial bast fibers [240,241,242,243]. Ramie fiber/sugar palm fiber reinforced epoxy hybrid composites were manufactured using a combination of melt mixing and injection molding techniques as shown in Figure 12 [45]. Numerous ramie fiber/PP composites were manufactured by changing the fiber length, content, and pretreatment technique. Increments in fiber length and content were associated with significant increases in tensile, flexural, and compression strengths. Nonetheless, they negatively affected the elongation behavior and impact strength of composites. The preparation of thermoplastic biodegradable composites containing ramie fibers and a PLA/PCL matrix was carried out via in situ polymerization [116]. The influences of fiber content and length on the impact and tensile strengths of this biodegradable composite reinforced with natural fibers were studied along with the effect of a silane coupling agent towards improving interfacial adhesion. Tensile and impact strengths were found to be highest with the use of a silane coupling agent, ramie fiber length of 5–6 mm, and 45 wt.% fiber content.

When compared to other natural fibers, the use of ramie fibers as reinforcement in hybrid composites is favored due to their superior mechanical qualities. Romanzini et al. [117] investigated the changes in chemical composition and thermal stability of ramie fibers post washing with distilled water. Apart from this, research on glass and washed ramie fiber composites was carried out, with an emphasis on the effects of using different fiber lengths (25, 35, 45, and 55 mm) and the fiber compositions, while the fiber loading was set at 21 vol.%. They reported that composites could be potentially produced from washed ramie fibers. The composite containing fiber length of 45 mm exhibited higher flexural strength despite the insignificant difference observed in lower volume fractions of glass fiber of 0:100 and 25:75. Better impact and flexural properties were also obtained from the increased glass fiber’s relative volume fraction up to a limit of 75% [117].

The major problem with employing natural fibers is that they are incompatible with a polymer matrix, which reduces the mechanical performance [244,245,246,247]. Tezara et al. [97] investigated the influence of stacking sequences, alkali treatment, and orientations of fiber on the mechanical characteristics of hybrid jute (J) and ramie (R) reinforced vinyl ester (VE) composites. First, woven fibers were made using three- and four-layer stacking sequences with a 0° orientation. A higher tensile strength value of 298.90 MPa was observed from the RJJR stacking sequence fabricated from different fiber orientations, e.g., 0°, 30°, 45°, and 90°. This was done to study the influence of fiber orientation on the flexural and tensile characteristics. 0° fiber orientation possessed significantly flexural and tensile strengths compared with other orientations of 28.90 MPa and 66.81 MPa, correspondingly. Enhancement of mechanical properties was also conducted via 5 wt.% and 10 wt.% alkali treatments, resulting in a maximum flexural strength (34.50%) increment in 0° RJJR with 5 wt.% compared with the untreated RJJR. They concluded that the fiber orientation and a lower alkali treatment concentration (5 wt.%) combination had significantly improved the mechanical characteristics of fiber hybrid composites.

Hand lay-up method employing epoxy as a matrix is used to manufacture bulletproof panels, where the prototype is more lightweight and economical compared to the conventional ones made of steel-based materials, Kevlar/aramid composite, and ceramic plates used in military antiballistic equipment [118]. The findings from bullet testing revealed the panels’ ability to resist high-impact projectile (level II) penetration and resulted in minimal fractures. However, level IV ballistic testing demonstrated the failure of all prototype panels to resist the projectile’s high-impact velocity. From the tests, ramie fiber has enough breaking strength and toughness to pass level II bullet testing. Among the matrices used to reinforce ramie fiber are included polyester [119], epoxy–bioresin [248], soy protein [249], epoxy [250] and PP [251].

### 3.10. Abaca/Banana Fiber Reinforced Hybrid Composites (ARPC)

The banana plant produces abaca/banana fiber, the strongest commercially available cellulose fiber, which is strong and seawater-resistant. Abaca is a native of the Philippines, where it is currently grown, as well as in Ecuador, and was then the most chosen rope fiber in marine applications.

**Figure 13:**
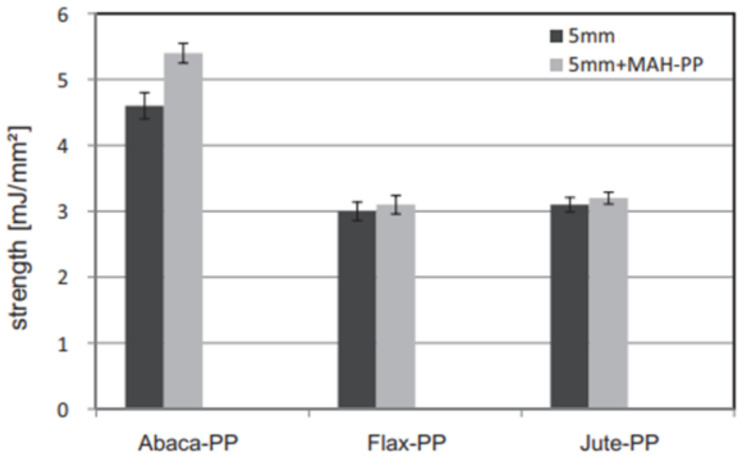
Notch Charpy strength of abaca/jute/flax fiber–PP composites comparison with and without MAH–PP. Reprinted with permission, Elsevier.

**Figure 14:**
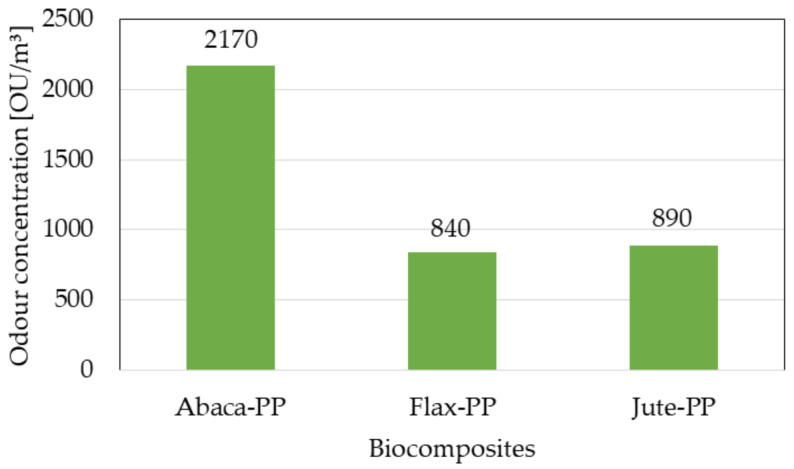
Comparison of odor emission concentration abaca/jute/flax fiber–PP composites. Reprinted with permission, Elsevier.

Bledzki et al. [120] studied the mechanical characteristics of abaca fiber reinforced PP composites with varying fiber lengths (5, 25, and 40 mm) and compounding procedures (mixer-injection, mixer compression, and direct compression moldings). When the length of the fibers was increased from 5 to 40 mm, the tensile and flexural characteristics were increased slightly, but not significantly. The mixer-injection molding technique outperformed the other two compounding procedures in mechanical performance (tensile strength was roughly 90% greater). The comparison of the composites of abaca fiber PP with the composites of jute and flax fiber PP revealed that the best falling weight impact properties and notched Charpy (Figure 13) were possessed by abaca fiber composites. Figure 14 shows the higher odor concentration of abaca fiber composites compared to flax and jute fiber composites.

The effects of fiber loading, frequency, and temperature on the polarity of banana fiber reinforced polyester composites were studied in a dynamic mechanical analysis [252]. The composites’ storage modulus at 40% fiber loading was the greatest, showing that the inclusion of abaca fiber in the polyester matrix resulted in reinforcing effects at higher temperatures. Enhanced fiber and matrix interactions were confirmed by the increased dynamic modulus and low damping values. Abaca fibers were reinforced with the matrices of cement [121], polyurethane [253], aliphatic polyester resin [254], PP [122,255], urea-formaldehyde [256], PE [123,124], polyester [125,126], and polyvinyl alcohol [127] to evaluate the properties of the produced composites.

### 3.11. Sisal Fiber Reinforced Hybrid Composites (SRPC)

Sisal is a type of *agave* (*Agave sisalana*) mostly grown in Brazil and East Africa. Between 1998 and 2010, global demand for sisal fibers and products was predicted to fall by 2.3% each year. Synthetic replacements and harvesting systems adoption that use less or no twine continued to undercut the conventional market for fibers. Sisal fiber will be used to make a wide range of non-structural and structural industrial goods using various polymer matrices.

The composites’ mechanical properties are heavily impacted by the bonding between the fiber and matrix, as reported by Senthilkumar et al. [257] and Ilyas et al. [258]. Good interfacial bonding induces transfer of the applied stress by the reinforced polymer composites to fibers. The hydrophilicity and hydrophobicity of the fibers and resin, respectively, usually result in poor bonding of the plant-based fibers that could be overcome via mechanical interlocking, chemical, inter-diffusion and electrostatic bondings, chemical pretreatment, as well as coupling agent [259]. Compression molding (CM), resin transfer molding (RTM), and injection molding are among the common techniques of natural fiber composite fabrication [260,261,262]. These methods differ from each other in terms of processing temperature, pressure, and speed. Sreekumar [263] studied the mechanical properties of the fabricated sisal fiber polyester composites via resin transfer and compression moldings. The RTM composites demonstrated a higher Young’s modulus, tensile and tensile flexural strengths, and flexural modulus. CM composites, on the other hand, possessed more water absorption and voids due to the weaker adhesion of fiber-matrix compared to RTM composites.

**Figure 15:**
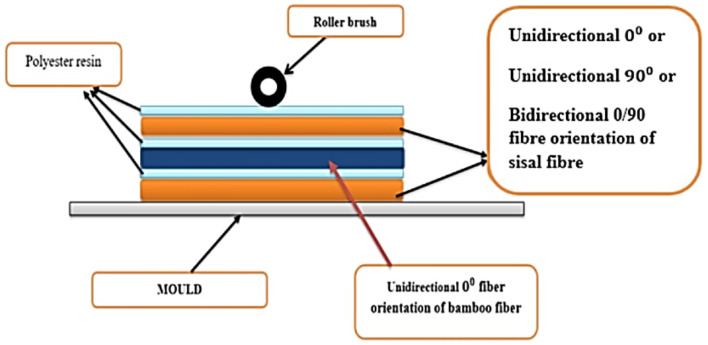
Fabrication of composite with varied orientations of sisal fiber [91]. Reprinted with permission, Elsevier.

Getu et al. [91] reported that composite materials possessed a low density with a high strength to weight ratio, stiffness to weight, strength ratios, and fatigue strength to weight ratio than conventional engineering materials, allowing them to be used in wide structural constructions applications. Lightweight natural fibers produce lightweight composite materials that in automotive applications improve fuel economy by minimizing harmful emissions. As shown in Figure 15, Getu et al. [91] prepared and characterized the performance of sisal and bamboo reinforced polyester hybrid composite (BSFRHC) with various sisal and unidirectional (UD) bamboo fiber orientations. Following that, BSFRHC was developed with a total fiber volume percentage of 20% via hand lay-up method using 3:1 bamboo to sisal fibers ratio prior to compressive, tensile, flexural and impact tests. It was concluded that varying fiber orientation resulted in variation in tensile strength; a higher tensile strength was found in the composite of bamboo/sisal fiber with 0° fiber orientation. The 0° fiber orientation composite demonstrated a higher compressive strength than the 90° fiber orientation composite and the bidirectional (0°/90°) fiber orientation composite. Higher tensile and flexural strengths were observed in the unidirectional 90° fiber orientation, whereas almost similar tensile strengths were obtained from the unidirectional 90° and bidirectional (0°/90°), and bidirectional (0°/90°) possesses higher flexural strength compared to unidirectional 90° fiber orientation. ANSYS Software was used to carry out the impact analysis of BSFRHC based vehicle internal door panel and the potential for the applications of interior automotive parts was revealed from the composites of sisal and bamboo fibers in unidirectional 0°.

Asaithambi et al. [129] conducted a study on the effect of Benzoyl Peroxide (BP) fiber surface treatment towards the mechanical characteristics of banana/sisal fiber (BSF) reinforced PLA composites [129]. BSF underwent BP treatment for the purpose of improving fiber and matrix adhesion. Twin-screw extrusion of BSF (30 wt.%) reinforced PLA (70 wt.%) hybrid composites was performed, followed by injection molding. The findings revealed that treated BSF possessed better bonding and wettability, resulting in the PLA matrix’s restricted motion. When comparing the composites of BSF-reinforced PLA with untreated BSF reinforced PLA and virgin PLA, the mechanical characteristics, e.g., flexural and tensile moduli, were improved.

Noorunnisa Khanam et al. [130] investigated the fluctuation of mechanical characteristics, e.g., flexural and tensile properties of the hybrid composites comprising randomly oriented unsaturated polyester-based sisal/carbon fibers varying fiber weight ratios by the hand lay-up approach. These hybrid composites were tested for chemical resistance to different solvents, acids, and alkalis. The influence of treating sisal fibers with NaOH on the tensile, flexural, and chemical resistance characteristics of these sisal/carbon hybrid composites was also investigated. The flexural and tensile characteristics of the hybrid composites were improved with the rising loading of carbon fiber, where the tensile and flexural characteristics of these hybrid composites were found to be superior to those of the matrix. Alkali treatment resulted in significant improvements in the tensile and flexural characteristics of the sisal/carbon hybrid composites. All compounds, excluding carbon tetrachloride, were resistant to these untreated and alkali-treated hybrid composites in chemical resistance tests.

Incorporation of zinc borate and magnesium hydroxide into sisal/PP composites as flame retardants was performed to improve the composites’ thermal stability as well as to reduce the composites’ burning rate [264]. The same study reported no synergistic effect from incorporating magnesium hydroxide and zinc borate into sisal/PP composites. Furthermore, at high shear rates, the sisal/PP composites showed substantial changes in shear viscosity, showing that the flame retardants utilized in this investigation did not affect the composites’ processability. The sisal/PP composites that had flame retardants added to them had tensile and flexural properties comparable to those of the sisal/PP composites without flame retardants.

Environmental impacts of degradation behavior [265], coupling agent influence on abrasive wear qualities, and the ageing effect [266] on mechanical characteristics have all been examined with sisal/PP composites. All plant fiber composites were developed by Zhang et al. [267] by transforming wood flour using a proper benzylation procedure and compounding of both discontinuous and continuous sisal fibers to create composites from renewable resources. The developed sisal/plasticized wood flour composites were found to be fully biodegradable from the degradation tests. The process of decomposition was accelerated by taking into account both lignin and cellulose in the composites. When it comes to practical applications, composites’ hydrophobicity and flame resistance are vital; therefore, molecular modification and/or integration of inorganic additives are appropriate approaches as long as the composite’s biodegradability is not compromised.

Many studies were performed on the composites of sisal fiber reinforced polyester concerning their characteristics of moisture absorption [268], as well as treatment of fiber with admicellar [269]. A few investigations were conducted on the composites of sisal fiber-reinforced phenolic resin, e.g., chemical alteration of such with lignins [270], hydroxyl-terminated polybutadiene rubber modification [271], cure cycles effect [272], employing glyoxal from natural resources [272], and alkali treatment effect [273]. Nevertheless, epoxy resin was employed as a matrix for sisal fiber-reinforced composites, and the effects of fiber orientation on electrical characteristics [274] and reinforcing degree [275] were investigated. A different matrix (cement) was also used in the sisal fiber-reinforced composites to study their cracking micro-mechanisms [276] and the influence of accelerated carbonation on cementitious roofing [277].

Towo et al. [278] prepared composites using treated sisal fibers with epoxy and polyester resin matrices. Dynamic thermal analysis and fatigue evaluation tests were conducted on the produced composites and revealed better mechanical characteristics in alkali-treated fiber bundle composites than untreated fiber bundle composites. The polyester resin matrices were most affected by alkali treatment, where improvements in the composites’ fatigue lives were found for the alkali-treated sisal fiber bundles. The superiority of alkali-treated fiber composites was analyzed and was associated with low cycle fatigue. Epoxy matrix composites possessed a longer fatigue life than polyester matrix composites. The chemical treatment had significantly and positively impacted the fatigue life of polyester matrix composites; however, it demonstrated a lesser effect on epoxy matrix composites. Studies on sisal fibers were also conducted with other matrices, e.g., rubber [279], phenol-formaldehyde [256], cellulose acetate [280], bio polyurethane [281], and polyethylene [282] in terms of their morphological, mechanical, cure, and chemical properties.

## 4. Mechanical Properties of NF Reinforcement Hybrid Composites

Researchers have been focusing their research interests on composites of natural fibers, e.g., biocomposites made of natural or synthetic resins reinforced by natural fibers. Natural fibers have numerous advantages, including their low density, which results in comparatively lightweight composites having excellent specific properties [244,283,284]. Additionally, these fibers offer significant cost savings and ease of processing, as well as being a highly renewable resource, thereby reducing reliance on domestic and foreign petroleum oil. Researchers have reviewed recent advances in natural fiber (e.g., flax, hemp, jute, kenaf, straw, bamboo, and coir) applications in composites [8,285,286]. Nilza et al. [287] designed and manufactured composites from three Jamaican natural cellulosic fiber: sugar cane bagasse, banana trunk, and coconut husk coir. The prepared samples were tested for carbon and ash contents, moisture content, water absorption, elemental and chemical analyses, and tensile strength.

### 4.1. Tensile Properties

Natural fiber-reinforced composites possess similar mechanical characteristics to synthetic fibers, as reported by Van De Velde and Kiekens [288] for hemp, flax, sisal, and jute fibers, in terms of strength and modulus compared to glass fiber. Srinivasan et al. [289] researched the ultimate tensile strength of the composites of glass fiber and banana/flax reinforced polymer (GFRP). A higher ultimate tensile strength (39 N/mm^2^) was observed in the flax banana-GFRP hybrid composite compared to the banana-GFRP and flax-GFRP composites with 30 N/mm^2^ and 32 N/mm^2^, respectively. Paul et al. revealed the mechanical characteristics of the composites of kenaf reinforced polypropylene showing improvements in ultimate tensile stress and tensile modulus with a rising fraction of fiber weight [23]. Table 3 displays the tensile properties comparison of different natural fibers with synthetic fibers.

### 4.2. Flexural Properties

The potential of composite materials’ use in structural applications is determined via a few parameters. Major strength is the flexural properties that include flexural strength, modulus, and load as well as deflection at the break. Flexural strength is related to the fiber content/fiber length, as reported in a few studies. Satyanarayana et al. [293] demonstrated that improvements in toughness and ductility of bamboo-mesh reinforced cement composites as well as significant enhancements in the tensile, flexural, and impact strengths. Banana and glass fibers were fabricated at different fiber lengths and loadings in the phenol-formaldehyde composites, and the mechanical properties were compared. From the composites of flexural property analysis, the optimum length of fiber needed for banana and glass fibers was different from the phenol-formaldehyde resole matrix reinforcement [295]. Aziz et al. observed the influence of random and long kenaf and hemp fibers alkalization and alignment on the formed composite fabricated via a combination of the fibers with polyester resin hot-pressed [296]. Long and alkalized fiber composites exhibited higher flexural strength and modulus compared with the as-received fiber composites. The decline in the flexural properties was due to water absorption that damaged and degraded fiber-matrix interfacial bonding; however, the maximum strain was simultaneously increased [297,298,299].

**Figure 16:**
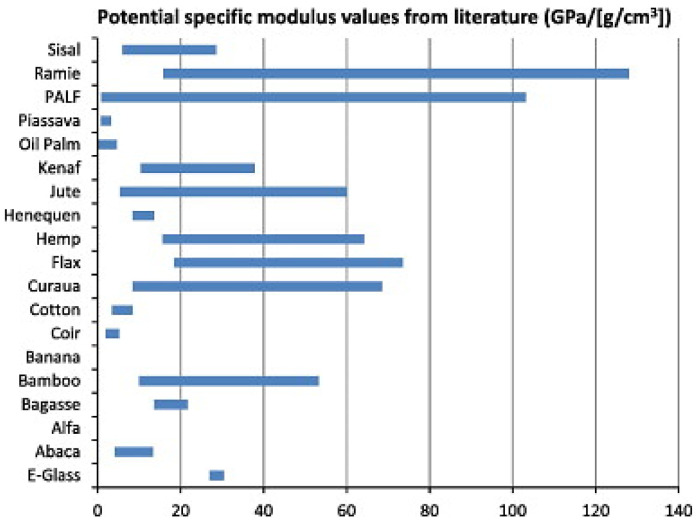
Comparison of values and ranges of potential specific modulus between natural and glass fibers [299]. Extracted with permission from Elsevier.

Shibata and team reported that the densified structure of kenaf fibers contributes to their composites’ higher flexural strength compared to the porous bagasse fibers [300]. Other researchers studied the effect of hybridizing the composites of jute/glass-reinforced epoxy on their mechanical properties. The E-glass fabric layers added to the composites’ outer layers revealed improvements in the properties of bending, tensile, and impact of the jute-reinforced composites [98]. A summary of specific moduli of natural and glass fibers is presented in Figure 16.

### 4.3. Impact Properties

Pothan et al. [301] examined the composites prepared from short banana fiber reinforced polyester, with the aim of studying the influence of fiber lengths and content on the composites’ impact strength. A 40 mm fiber length yielded the highest impact strength, while 40% incorporation of untreated fibers resulted in a 34% improvement of impact strength. Another study on the impact behavior of 35% jute/vinyl ester composites reinforced with alkali-treated and untreated fibers revealed hemicellulose removal, improving the crystallinity and, consequently, better fiber distribution [302]. Sanjay et al. [303] compared different compositions of laminates to investigate the impact behavior of the composites’ banana/E-glass fabrics reinforced polyester hybrid and found 6 J impact strength in the hybrid laminate, which was the highest value attained.

### 4.4. Hardness Properties

**Figure 17:**
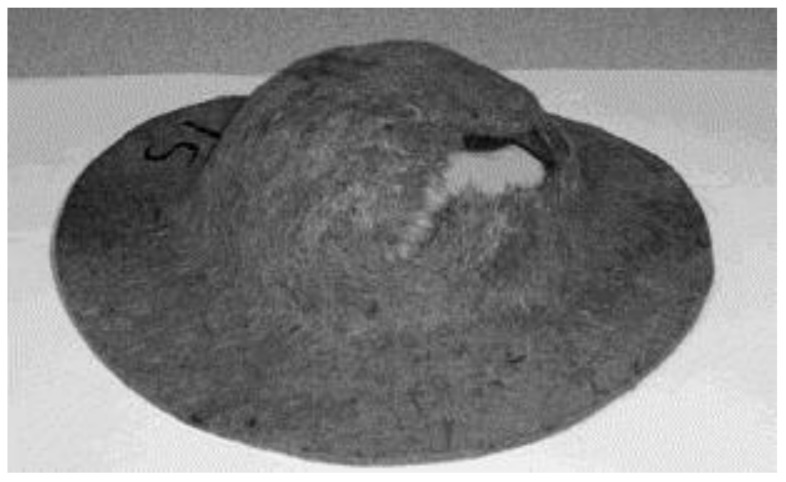
Kenaf–PP/MAPP composite hybrid composites tested for hardness properties [304]. Extracted with permission.

Zampaloni et al. [304] discussed the excellent potential of the current materials substitute by the Kenaf–maleated polypropylene composites that demonstrated more efficient modulus/cost as well as better specific strength and modulus at a cheaper cost compared to the materials reinforced with E-glass, coir, and sisal (Figure 17). The hardness of various laminates fabricated from hybrid composites of banana/E-glass fabrics reinforced polyester utilizing different stacking sequences was measured by Sanjay et al. [303] Laminate L1, or the composite of pure glass fiber, possessed the hardness of 26.72 HV, while laminate L2 (composites of pure banana fiber) exhibited the poorest hardness of 12.36 HV. 

## 5. Current Application on NF Reinforcement Hybrid Composites

Due to its low manufacturing cost, max strength ratio, and simple manufacturing process, hybrid natural fiber composites have already been widely extensively utilized in numerous textile and engineering applications. Furthermore, natural fiber composites demonstrated a good combination of mechanical qualities for aerospace and automotive applications, including its enhanced impact strength, tensile, bending and compressive behavior, as well as improved fatigue properties. Bio-based hybrid composites are a rapidly increasing product in the industrial sectors as a means of reducing environmental effects in today’s society.

### 5.1. Automotive

The automotive industry demands composite materials in order to comply with new regulations and to remain competitive. At present, plant fibers are used in the exterior and interior components in semi- or non-structural applications, fulfilling the performance standards, e.g., elongation, ultimate breaking force, impact strength, flexural properties, flammability, fogging characteristics, acoustic absorption, odor, dimensional stability, aptness for processing dwell time and temperature, crash resistance, and water absorption. A few renowned automakers, e.g., Volkswagen-Audi, Daimler-Chrysler, and Opel-GM, have already begun incorporating natural fiber composites into their passenger car parts, including rear parcel shelf, door trim panels, and seat squabs [305,306]. Table 4 shows the present use of natural fiber in the automobile sector by the big automobile companies.

The prospects for a lightweight design from plant fiber composites are demonstrated, for instance, in Mercedes E-Class’s panels and even external underbody panels and Volkswagen’s door structures (phenol-formaldehyde/flax composite) [307]. Given the unique qualities of natural fiber composites, an approximately 15% weight reduction in components is feasible compared to glass fiber reinforced composites.

### 5.2. Aerospace

During the early stages of the aircraft industry’s development, aircraft structures were invented using wire, wood (natural composite), and fabric compositions. Aluminum alloys have been the dominating material in the aerospace industry since the 1930s. The newest components of civil aircraft are made of natural fibers that are also used as a substructure in conjunction with fibers containing composites and other synthetic fibers, e.g., glass, carbon, and Kevlar. The design of V22 Osprey tiltrotor’s wings is extremely rigid and risky, and is most likely to be constructed using fiber composites with low-density materials. In defense aircraft, a fascinating advancement called “stealth” has emerged, which requires the designer to achieve the smallest possible radar cross-section (RCS) by reducing the potentials of early detection via defending radar sets. Constant radius changes are required to create the airframe’s essential compound curvatures, which are much easier to build using composites compared to metal and radar-absorbent material (RAM).

### 5.3. Oil and Gas

Hybrid natural fiber-reinforced composites of natural fiber-reinforced composites have been found to have less critical environmental impacts than glass fiber reinforced composites in some applications [309]. Natural fibers have been used with glass fiber for underground pipes; this application is faster and offers adequate strength. However, certain issues, e.g., water absorption and strength, have yet to be studied [310].

### 5.4. Maritime

According to Moreau et al. [311], fiber-reinforced plastic (FRP) structures in boat construction uses primarily thermosetting resins (e.g., vinyl ester, epoxy, polyester, etc.) Only lately have thermoplastic resins (polypropylene, polyamide, PBT, PET, etc.) started being applied in fittings or boat-building. In recent times, the resin structure has evolved in 2 forms: both low styrene content and emission are commercially available despite the evolution of bio-based resins; however, conventional resins are still relevant in the nautical area [174].

The environmental benefits of adhesives and bio-based resins are found in their elimination of toxins in common, their emphasis on human health and the environment, their reduction of hazardous as well as toxic materials and waste, recycling capabilities, and the decline of polluting air emissions. Natural fibers are also gaining popularity in the composites sector. However, their application in structural components is limited due to their generally poor physical characteristics, whereas at present, they are applied in filling functions. Glass fibers provide for 89% of the fiber’s capacity used in composites on a global scale. In contrast, natural fibers account for just 10%. Simultaneously, several R&D studies of natural fiber uses are underway, resulting in more industrial uses in the foreseeable future.

Glass fibers were accessible during World War II, shortly after polyester resins were created, as a result of the accidental discovery of a manufacturing method involving blown air on a molten glass stream. Soon after, glass-reinforced plastic became popular, and in the early 1950s, GRP boats became accessible. Fiberglass boats are significantly more appealing due to their strength, including their high vibration damping ability, lightweight, low construction costs, high impact resistance, ease of fabrication, maintenance, and repair.

Fiberglass boat construction has evolved over the years to include a variety of means with the sole objective of an improvement in boat construction skills and techniques to fulfill the aims of fiberglass boat building, e.g., to produce lightweight products, corrosion and impact resistance, vibration damping, low cost, and ease of construction. Now, hybrid natural fiber (NF) composites are broadly utilized in a variety of technical applications that comprise marine applications, particularly in boat construction.

### 5.5. Textiles

Pineapple leaves (PAL) have been utilized as threads and fabrics in a variety of nations for centuries. Excellent pina fibers are derived from pineapple leaves in the Philippines and are used to create textiles for table linens, dresses, mats, bags, and other apparel items. Applications of PAL in the textile industry are established in Indonesia, while in Malaysia, the efforts in PAL employment in Malaysia are still in their infancy. The PAL dresses, however, are recognized to be expensive, which is understandable because of the tedious procedures involved.

## 6. Estimated Costing for NF as Reinforcement in Hybrid Composites

Excellent specific properties and cheap natural fiber composites are the primary factors of their attractiveness for wide applications, as reported by Sanjay et al. [303] and Ho et al. [292] The price lists of NF, synthetic fibers, and matrices in the US and Malaysia currencies, USD and MYR, respectively, are tabulated in Table 5. Table 6 tabulated the estimated cost for hybrid NF/synthetic fibers and matrices for numerous applications.

## 7. Conclusions and Future Directions

Currently, the production of many bio-based plastics has been demonstrated at the demo and pilot scale, and some had been hugely commercialized. Some of the products are partly bio-based (i.e., polyamides, polyols bio-based polyethylene (PE), polypropylene (PP), or polyethylene terephthalate (PET)), and some of them are entirely new plastics (i.e., thermoplastic starch (TPS), polyhydroxybutyrate (PHB), polyhydroxyalkanoates (PHAs), or poly (lactic acid) (PLA). Partly bio-based plastics often require petrochemical monomers that cannot be resembled by bio-based substitutions, at least not at a reasonable price. Today’s bio-based plastics are sophisticated materials that could technically be a substitute for around 90% of the plastics we use today.

Natural fiber reinforced hybrid composites are superior to petroleum-based composites because they have a higher strength-to-weight ratio, a low manufacturing cost due to their facile processes, and are environmentally beneficial. As a result, natural fiber composites have numerous advantages in commercial, industrial and engineering applications. Natural fibers, on the other hand, have lower strength than synthetic composites, but when combined with synthetic or biosynthetic composites, they offer high strength with a lower environmental impact. This study outlines the properties of natural fiber, composite hybridization, estimated costing, and natural fiber applications in various industries. This gives a comprehensive idea of how natural fibers are processed and commercialized. The characteristics of natural fibers such as sisal, jute, abaca, sugar palm, kenaf and hemp, were studied. In addition, the numerous applications of the hybrid composites in various sectors were described.

## Figures and Tables

**Table 2:** Natural fiber reinforced hybrid composites.

Natural Fiber	Matrix	Hybrids	Process	Ref.
**Sugar palm fiber (SPF)**				
Sugar palm fiber	Unsaturated polyester	Woven glass	Compression molding	[28]
Sugar palm fiber	Unsaturated polyester	Glass fiber	Hand lay-up	[33]
Sugar palm fiber	Thermoplastic polyurethane	Glass fiber	Melt compounding	[34]
Sugar palm yarn fiber	Epoxy	Carbon fiber	Hand lay-up	[35]
Benzoyl treated sugar palm fiber	Epoxy	Glass fiber	Hand lay-up	[36,37]
Sugar palm fiber	Thermoplastic sugar palm starch/agar	Seaweed fiber	Hydraulic thermo-press	[38]
Sugar palm fiber	Thermoplastic polyurethane	Roselle fiber	Hot press	[39]
Sugar palm fiber	Cornstarch	Cornhusk	Solution casting	[40]
Sugar palm yarn fiber	Unsaturated polyester	Glass fiber	Sheet molding	[41]
Sugar palm fiber	Cassava starch	Cassava fiber	Casting	[42]
Sugar palm fiber	Polypropylene	Kenaf fiber	Compression molding	[43]
Sugar palm fiber	Cornstarch	Cornstalk fiber	Solution casting	[44]
Sugar palm fiber	Epoxy	Ramie fiber	Compression molding	[45]
Sugar palm fiber	Vinyl ester	Roselle fiber	Hand lay-up	[46]
Sugar palm fiber	Polypropylene	Glass fiber	Film stacking and hot compression	[47]
**Kenaf fiber (KF)**				
Kenaf fiber	Epoxy	Glass fiber	Sheet molding	[48]
Kenaf fiber	unsaturated polyester (UPE)	Glass fiber	Sheet molding	[49]
Kenaf fiber	Epoxy	Silica	Hand lay-up	[50,51]
Kenaf fiber	Epoxy	Bamboo fiber/nanoclay	Hand lay-up	[52]
Kenaf fiber	Epoxy	Oil palm/montmorillonite	Hand lay-up	[53]
Kenaf fiber	Polypropylene-grafted maleic anhydride (PP-g-MA)	Graphene nanosheets	Hot press	[54]
Kenaf core	Polypropylene	Bleached nanocellulose	Melt mixing compounding	[55]
Kenaf fiber	Epoxy	Glass fiber	Filament winding	[56]
Kenaf fiber	Epoxy	Pet yarn	Cold press	[57]
Kenaf fiber	Polyethylene terephthalate	Glass fiber	Compression molding	[58]
Kenaf fiber	Epoxy	Kevlar	Hand lay-up	[59]
Kenaf fiber	Polyester	Banana fiber	Hand lay-up	[60]
Kenaf fiber	Indian almond fiber	Kenaf fiber	Hand lay-up	[61]
Kenaf fiber	Epoxy	Glass/waste tea leaf fiber	Compression molding	[62]
Kenaf fiber	Epoxy	Oil palm fiber	Hand lay-up	[63]
Woven kenaf fiber	Polypropylene	Glass fiber	Hot press molding	[64]
Kenaf fiber	Polypropylene	E-glass	Hot compression molding	[65]
Kenaf fiber	Epoxy	Bamboo fiber	Hand lay-up	[66]
Kenaf fiber	Polypropylene	Wood flour	Injection molding	[67]
	Polylactic acid (PLA)	Kenaf	Fused Deposition Modeling (FDM)	[68]
**Oil palm empty fruit bunches fiber (OPEFB)**				
Oil palm empty fruit bunches fiber	Epoxy	MgO_2_ pet yarn	Compression molding	[69]
Oil palm empty fruit bunches fiber	Polyester resin	MgO_2_ pet yarn	Compression molding	[69]
Oil palm empty fruit bunches fiber	Epoxy	Woven kenaf fabric	Hand lay-up	[70]
Oil palm empty fruit bunches fiber	Polypropylene (PP) matrix		Injection molding	[71]
Oil palm empty fruit bunches fiber	Phenolic formaldehyde (PF) resin	Sugarcane bagasse (SCB) fiber	Hand lay-up	[72]
Oil palm empty fruit bunches fiber	Resin	Gamma-irradiated kevlar	Hand lay-up	[73]
Oil palm empty fruit bunches fiber	Recycled polypropylene (RPP)	Glass fiber	Extrusion and injection molding	[74]
Oil palm fibers	Polyester resin	Chopped strand mat (CSM) glass fibers	Hybrid laminates	[75]
Oil palm empty fruit bunches fiber	Polypropylene	Glass fiber	Hot pressing	[76]
**Pineapple leaf fibers (PALF)**				
Pineapple leaf fibers	Carbon hybrid laminate		Vacuum infusion	[77]
Pineapple leaf fibers	Polylactic acid (PLA)	Alkali treated coir	Compression molding	[78]
Pineapple leaf fibers	Vinyl ester	Glass fiber	Automated spray-up	[79]
Pineapple leaf fibers	Polyester	Banana/glass fiber	Hand lay-up	[80]
Silane treated pineapple leaf fiber	Phenolic hybrid	Kenaf fiber	Hydraulic pressure hot press	[81]
Pineapple leaf fibers	Polyester	Sisal fiber	Injection molding	[82]
**Bamboo fiber (BF)**				
Long bamboo fiber	Epoxy		Compression molding	[83]
Short bamboo fiber	POlypropylene	Glass fiber	Injection molding	[84]
Bamboo fiber	Maleic anhydride grafted polypropylene (MAPP)	Glass fiber	Injection molding	[85]
Bamboo fiber	Polypropylene	Glass fiber	Compression molding	[86,87]
Bamboo fiber	Epoxy	Ceramic fillers	Compression molding	[88]
Bamboo fiber	Epoxy polymer	Jute fiber	Hand lay-up	[89]
Bamboo fiber	Epoxy	Flax fiber mat	Hand lay-up	[90]
Bamboo fiber	Epoxy	Sisal fiber	Hand lay-up	[91]
Bamboo fiber	Epoxy	Cotton yarn	Compression molding	[92]
Bamboo leaf fiber ash	Aluminium metal matrix	Rice husk ash	Hand lay-up	[93]
Bamboo fiber	Epoxy	Kenaf fiber	Hand lay-up	[66]
**Jute fiber (JF)**				
Alkali treated jute fiber	Vinyl ester resin		Compression molding	[94]
Jute fiber	Epoxy	Carbon fiber	Hand lay-up	[95]
Jute fiber	Epoxy polymer	Bamboo fiber	Hand lay-up	[89]
Woven jute	Polyester	Glass fabric	Hand lay-up	[96]
Woven jute	Vinyl ester	Ramie fiber	Hand lay-up	[97]
Jute fiber	Epoxy resin	Glass fiber	Resin infusion	[98]
Jute fiber	Polyester	Glass fiber	Injection molding	[99]
Jute fiber		Hemp/Flax fiber	Hand lay-up	[100]
Jute fiber	Polyester	Cotton woven fabric	Hand lay-up	[101]
Jute fiber	Polyester	Woven fabric basalt fiber	Compression molding	[102]
**Hemp fiber (HF)**				
Alkaline-treated hemp fiber	Polyester resin	Carbon fiber	Hand lay-up	[103]
Hemp fiber mat	Green epoxy	Sisal fiber	Hand lay-up method and hot press	[104]
Hemp fiber	Unsaturated polyester	Soybean oil/nanoclay	Compression molding	[105]
Hemp fiber	Polylactic acid	Sisal fiber	Injection molding	[106]
Hemp fiber	HDPE	Basalt fiber	Injection molding	[107]
Interwoven hemp fiber	PET		Vacuum infusion	[108]
**Flax fiber (FF)**				
Flax fiber	Epoxy	Hemp fiber	Hand lay-up	[100]
Flax fiber	Epoxy	Jute/hemp fiber	Hand lay-up	[100]
Flax fiber	Vinyl ester	Glass fiber	Resin infusion	[109]
Short flax fiber	Polypropylene		Injection molding	[110]
Flax fiber	Polypropylene	Kenaf/hemp fiber	Compression molding	[111]
Flax fiber	Polylactic acid	Kenaf/hemp fiber	Compression molding	[111]
Flax fiber	Epoxy		Vacuum infusion	[112]
Flax fiber	Vinyl ester	Basalt fiber	Vacuum assisted resin infusion	[113]
Flax fiber	Epoxy resin	Glass fiber	Compression molding	[114]
Flax fiber	Vinyl ester	Glass fiber	Resin infusion	[109]
Flax fiber	Barium sulphate	Woven aloevera	Compression molding	[115]
**Ramie fiber (RF)**				
Ramie fiber	Polylactic acid	Poly(ε-caprolactone)	Compression molding	[116]
Ramie fiber	PVA	Glass fiber	Compression molding	[117]
Ramie fiber	Vinyl ester	Jute fiber	Hand lay-up	[97]
Ramie woven	Epoxy		Hand lay-up	[118]
Ramie cloth	Unsaturated polyester resin		Resin casting	[119]
**Abaca/banana fiber (ABF)**				
Abaca/banana fiber	Polypropylene		Mixer-injection, mixer compression, and direct compression moldings	[120]
Abaca fiber	Cement	Silica		[121]
Enzyme modified abaca fiber	Polypropylene		Injection molding	[122]
Abaca fiber	Polyethylene	Banana fiber	Rotational molding	[123]
Banana fiber	Low density polyethylene		Compression molding	[124]
Abaca fiber	Polystyrene		Compression molding	[125]
Plain weave abaca fiber	Polyester resin		Hand lay-up	[126]
Banana fiber	Polyvinyl alcohol resin		Hand lay-up	[127]
**Sisal fiber (SF)**				
Sisal fiber	Phenolic resin	Aramid fiber	Compression molding	[128]
Sisal fiber	Bioepoxy	Hemp fiber	Hand lay-up	[104]
Sisal fiber	Polyester	Bamboo fiber	Hand lay-up	[91]
Sisal fiber	PLA	Banana fiber	Injection molding	[129]
Sisal fiber	Unsaturated polyester	Carbon fibers	Hand lay-up	[130]
Sisal fiber	Waste carbon	Glass fiber	Single extrusion and press consolidation	[131]
Sisal fiber	Epoxy	Jute fiber	Hand lay-up	[132]

**Table 3:** Tensile properties of natural and inorganic fibers.

Fibers	Density (kg/m^3^)	Diameter (μm)	Tensile Strength (MPa)	Tensile Modulus (GPa)	% Elongation	Ref.
Sugar Palm	1290	99–311	190.29	3.69	19.6	[157]
Jute	1460	-	393–800	10–30	1.5–1.8	[290]
Sisal	1450	50–300	227–400	9–20	2–14	[290]
Kenaf	1400	81	250	4.3	-	[290]
Flax	1500	-	345–1500	27.6–80	1.2–2.3	[291]
Hemp	1480	-	550–900	70	1.6	[291]
Banana	1350	80–250	529–759	8.20	1–3.5	[292]
Coir	1150	100–460	108–252	4–6	15–40	[292]
Bamboo	910	-	503	35.91	1.4	[292]
Cotton	1600	-	287–597	5.5–12.6	3–10	[293]
E-glass	2550	<17	3400	73	3.4	[294]
S-glass	2500	-	4580	85	4.6	[294]
Carbon (Std. PAN-based)	1400	-	4000	230–240	1.4–1.8	[294]

**Table 4:** Lists of natural fibers reinforced hybrid composites currently being used in the automotive industry. Extracted from Ref. [308] with permission.

Automobile Model	Natural Fiber Utilized	Applications
Audi A2	Flax, sisal fibers and polyurethane	Door trim panels
BMW 7 Series	Sisal fiber	Door trim panels
Chevrolet Impala	Flax fiber and polypropylene	Rear shelf compartment
Ford Focus and Fiesta	Kenaf fiber and sun	Interior door panels
Honda Pilot	Wood fiber	Floor area parts
Mercedes Benz A, C, E and S-class	Flax, hemp, sisal, cotton, abaca and jute fibers	Underbody panels, seat back rests, engine and transmission cover and rear panel shelves
Toyota Prius and Raum	Corn biopolymer, starch and kenaf fiber	Instrument panels, sun visors, ceiling surface skins and spare tire cover

**Table 5:** Estimated cost of natural, synthetic fiber and matrices in US Dollar (USD) and Malaysia Ringgit (MYR). Extracted from Ref. [292] with permission.

No.	Types of Natural Fiber	Cost (Money per Tons)	
		USD	MYR
1.	Bamboo	500	2092.80
2.	Banana	890	3725.18
3.	Flax	3150	13,184.64
4.	Hemp	1550	6487.68
5.	Jute	950	3976.32
6.	Kenaf	400	1674.24
7.	Pineapple	455	1904.45
8.	Sisal	650	2720.64
9.	Sugar palm	4000	16,742.40
**No.**	**Types of Synthetic Fiber**	**Cost (** **Money per Tons)**	
		**USD**	**MYR**
1.	Carbon	12,500	52,320.00
2.	Kevlar (aramid)	20,000	83,712.00
3.	Fiber Glass	980	4101.89
4.	Glass	1500	6278.40
**No.**	**Types of Matrices**	**Cost (** **Money per Tons)**	
		**USD**	**MYR**
1.	Epoxy	2650	11,091.84
2.	Polyester	550	2302.08
3.	Vinyl ester	1550	6487.68
4.	Polyurethane	2750	11,510.40

Currency United States Dollar (USD) to Malaysian Ringgit (MYR) on date 8 June 2021.

**Table 6:** Estimated costing for NF as reinforcement in hybrid composites (in US Dollar/USD and Malaysia Ringgit/MYR) [303].

No.	Reinforced Composite			Estimated Cost (Money per Tons)	
	Natural Fiber	Synthetic Fiber	Matrices	USD	MYR
1.	Bamboo	Carbon	Epoxy	15,650	65,504.64
2.	Bamboo	Carbon	Polyester	15,700	65,713.92
3.	Bamboo	Carbon	Vinyl ester	16,700	69,899.52
4.	Bamboo	Carbon	Polyurethane	17,900	74,922.24
5.	Bamboo	Kevlar	Epoxy	25,300	105,895.68
6.	Bamboo	Kevlar	Polyester	21,050	88,106.88
7.	Bamboo	Kevlar	Vinyl ester	22,100	92,501.76
8.	Bamboo	Kevlar	Polyurethane	23,250	97,315.20
9.	Bamboo	Fiber Glass	Epoxy	4130	17,286.53
10.	Bamboo	Fiber Glass	Polyester	2030	8511.59
11.	Bamboo	Fiber Glass	Vinyl ester	3030	12,704.49
12.	Bamboo	Fiber Glass	Polyurethane	4230	17,735.97
13.	Bamboo	Glass	Epoxy	4650	19,496.99
14.	Bamboo	Glass	Polyester	2550	10,691.90
15.	Bamboo	Glass	Vinyl ester	3550	14,884.80
16.	Bamboo	Glass	Polyurethane	4750	19,916.27
17.	Banana	Carbon	Epoxy	16,040	67,254.12
18.	Banana	Carbon	Polyester	13,940	58,449.03
19.	Banana	Carbon	Vinyl ester	14,940	62,641.93
20.	Banana	Carbon	Polyurethane	16,140	67,673.41
21.	Banana	Kevlar	Epoxy	23,540	98,700.87
22.	Banana	Kevlar	Polyester	21,440	89,895.78
23.	Banana	Kevlar	Vinyl ester	22,440	94,088.68
24.	Banana	Kevlar	Polyurethane	23,640	99,120.16
25.	Banana	Fiber Glass	Epoxy	4520	18,951.91
26.	Banana	Fiber Glass	Polyester	2420	10,146.82
27.	Banana	Fiber Glass	Vinyl ester	3420	14,339.72
28.	Banana	Fiber Glass	Polyurethane	4620	19,371.20
29.	Banana	Glass	Epoxy	5040	21,132.22
30.	Banana	Glass	Polyester	2940	12,327.13
31.	Banana	Glass	Vinyl ester	3940	16,520.03
32.	Banana	Glass	Polyurethane	5140	21,551.51
33.	Flax	Carbon	Epoxy	18,300	76,730.07
34.	Flax	Carbon	Polyester	16,200	67,924.98
35.	Flax	Carbon	Vinyl ester	17,200	72,117.88
36.	Flax	Carbon	Polyurethane	18,400	77,149.36
37.	Flax	Kevlar	Epoxy	25,800	108,176.82
38.	Flax	Kevlar	Polyester	23,700	99,371.73
39.	Flax	Kevlar	Vinyl ester	24,700	103,564.63
40.	Flax	Kevlar	Polyurethane	25,900	108,596.11
41.	Flax	Fiber Glass	Epoxy	6780	28,427.86
42.	Flax	Fiber Glass	Polyester	4680	19,622.77
43.	Flax	Fiber Glass	Vinyl ester	5680	23,815.67
44.	Flax	Fiber Glass	Polyurethane	6880	28,847.15
45.	Flax	Glass	Epoxy	7300	30,608.17
46.	Flax	Glass	Polyester	5200	21,803.08
47.	Flax	Glass	Vinyl ester	6200	25,995.98
48.	Flax	Glass	Polyurethane	7400	31,027.46
49.	Hemp	Carbon	Epoxy	16,700	70,021.43
50.	Hemp	Carbon	Polyester	14,600	61,216.34
51.	Hemp	Carbon	Vinyl ester	15,600	65,409.24
52.	Hemp	Carbon	Polyurethane	16,800	70,440.72
53.	Hemp	Kevlar	Epoxy	24,200	101,468.18
54.	Hemp	Kevlar	Polyester	24,750	107,967.18
55.	Hemp	Kevlar	Vinyl ester	26,300	110,273.27
56.	Hemp	Kevlar	Polyurethane	29,050	121,803.74
57.	Hemp	Fiber Glass	Epoxy	5180	21,719.22
58.	Hemp	Fiber Glass	Polyester	3080	12,914.13
59.	Hemp	Fiber Glass	Vinyl ester	4080	17,107.03
60.	Hemp	Fiber Glass	Polyurethane	5280	22,138.51
61.	Hemp	Glass	Epoxy	5700	23,899.53
62.	Hemp	Glass	Polyester	3600	15,094.44
63.	Hemp	Glass	Vinyl ester	4600	19,287.34
64.	Hemp	Glass	Polyurethane	5800	24,318.82
65.	Jute	Carbon	Epoxy	16,100	67,505.69
66.	Jute	Carbon	Polyester	14,000	58,700.60
67.	Jute	Carbon	Vinyl ester	15,000	62,893.50
68.	Jute	Carbon	Polyurethane	16,200	67,924.98
69.	Jute	Kevlar	Epoxy	23,600	98,952.44
70.	Jute	Kevlar	Polyester	21,500	90,147.35
71.	Jute	Kevlar	Vinyl ester	22,500	94,340.25
72.	Jute	Kevlar	Polyurethane	23,700	99,371.73
73.	Jute	Fiber Glass	Epoxy	4580	19,203.48
74.	Jute	Fiber Glass	Polyester	2480	10,398.39
75.	Jute	Fiber Glass	Vinyl ester	3480	14,591.29
76.	Jute	Fiber Glass	Polyurethane	4680	19,622.77
77.	Jute	Glass	Epoxy	5100	21,383.79
78.	Jute	Glass	Polyester	3000	12,578.70
79.	Jute	Glass	Vinyl ester	4000	16,771.60
80.	Jute	Glass	Polyurethane	5200	21,803.08
81.	Kenaf	Carbon	Epoxy	15,550	65,199.60
82.	Kenaf	Carbon	Polyester	13,450	56,394.50
83.	Kenaf	Carbon	Vinyl ester	14,450	60,587.40
84.	Kenaf	Carbon	Polyurethane	15,650	65,618.88
85.	Kenaf	Kevlar	Epoxy	23,050	96,646.35
86.	Kenaf	Kevlar	Polyester	20,950	87,841.25
87.	Kenaf	Kevlar	Vinyl ester	21,950	92,034.15
88.	Kenaf	Kevlar	Polyurethane	23,150	97,065.63
89.	Kenaf	Fiber Glass	Epoxy	4030	16,897.39
90.	Kenaf	Fiber Glass	Polyester	1930	8092.30
91.	Kenaf	Fiber Glass	Vinyl ester	2930	12,285.20
92.	Kenaf	Fiber Glass	Polyurethane	4130	17,316.68
93.	Kenaf	Glass	Epoxy	4550	19,077.69
94.	Kenaf	Glass	Polyester	2450	10,272.60
95.	Kenaf	Glass	Vinyl ester	3450	14,465.50
96.	Kenaf	Glass	Polyurethane	4650	19,496.99
97.	Pineapple	Carbon	Epoxy	15,605	65,430.20
98.	Pineapple	Carbon	Polyester	13,505	56,625.11
99.	Pineapple	Carbon	Vinyl ester	14,505	60,818.01
100.	Pineapple	Carbon	Polyurethane	15,705	65,849.49
101.	Pineapple	Kevlar	Epoxy	23,105	96,876.95
102.	Pineapple	Kevlar	Polyester	21,005	88,071.86
103.	Pineapple	Kevlar	Vinyl ester	22,005	92,264.76
104.	Pineapple	Kevlar	Polyurethane	23,205	97,296.24
105.	Pineapple	Fiber Glass	Epoxy	4085	17,128.00
106.	Pineapple	Fiber Glass	Polyester	1985	8322.91
107.	Pineapple	Fiber Glass	Vinyl ester	2985	12,515.81
108.	Pineapple	Fiber Glass	Polyurethane	4185	17,547.29
109.	Pineapple	Glass	Epoxy	4605	19,308.30
110.	Pineapple	Glass	Polyester	2505	10,503.21
111.	Pineapple	Glass	Vinyl ester	3505	14,696.11
112.	Pineapple	Glass	Polyurethane	4705	19,727.59
113.	Sisal	Carbon	Epoxy	15,800	66,247.82
114.	Sisal	Carbon	Polyester	13,700	57,442.73
115.	Sisal	Carbon	Vinyl ester	14,700	61,635.63
116.	Sisal	Carbon	Polyurethane	15,900	66,667.11
117.	Sisal	Kevlar	Epoxy	23,300	97,694.57
118.	Sisal	Kevlar	Polyester	21,200	88,889.48
119.	Sisal	Kevlar	Vinyl ester	22,200	93,082.38
120.	Sisal	Kevlar	Polyurethane	23,400	98,113.86
121.	Sisal	Fiber Glass	Epoxy	4280	17,945.61
122.	Sisal	Fiber Glass	Polyester	2180	9140.52
123.	Sisal	Fiber Glass	Vinyl ester	3180	13,333.42
124.	Sisal	Fiber Glass	Polyurethane	4380	18,364.90
125.	Sisal	Glass	Epoxy	4800	20,125.92
126.	Sisal	Glass	Polyester	2700	11,320.83
127.	Sisal	Glass	Vinyl ester	3700	15,513.73
128.	Sisal	Glass	Polyurethane	4900	20,545.21
129.	Sugar palm	Carbon	Epoxy	19,150	80,294.04
130.	Sugar palm	Carbon	Polyester	17,050	71,488.94
131.	Sugar palm	Carbon	Vinyl ester	18,050	75,681.85
132.	Sugar palm	Carbon	Polyurethane	19,250	80,713.32
133.	Sugar palm	Kevlar	Epoxy	26,650	111,740.78
134.	Sugar palm	Kevlar	Polyester	24,550	102,935.69
135.	Sugar palm	Kevlar	Vinyl ester	25,550	107,128.60
136.	Sugar palm	Kevlar	Polyurethane	26,750	112,160.07
137.	Sugar palm	Fiber Glass	Epoxy	7630	31,991.83
138.	Sugar palm	Fiber Glass	Polyester	5530	23,186.74
139.	Sugar palm	Fiber Glass	Vinyl ester	6530	27,379.64
140.	Sugar palm	Fiber Glass	Polyurethane	7730	32,411.12
141.	Sugar palm	Glass	Epoxy	8150	34,172.14
142.	Sugar palm	Glass	Polyester	6050	25,367.04
143.	Sugar palm	Glass	Vinyl ester	7050	29,559.94
144.	Sugar palm	Glass	Polyurethane	8250	34,591.42

Currency United States Dollar (USD) to Malaysian Ringgit (MYR) on date 8 June 2021.
